# Discriminating the occurrence of inundation in tsunami early warning with one-dimensional convolutional neural networks

**DOI:** 10.1038/s41598-022-13788-9

**Published:** 2022-06-20

**Authors:** Jorge Núñez, Patricio A. Catalán, Carlos Valle, Natalia Zamora, Alvaro Valderrama

**Affiliations:** 1 Departamento de Obras Civiles, Universidad Técnica Federico Santa María, Valparaíso, 2390123 Chile; 2 Departamento de Ciencia de Datos e Informática, Universidad de Playa Ancha Valparaíso, Valparaiso, Chile; 3 Computer Applications in Science and Engineering Department, Barcelona Supercomputing Center (BSC), 08034 Barcelona, Spain; 4 Universidad Técnica Federico Santa María, Valparaíso, Chile

**Keywords:** Natural hazards, Physical oceanography

## Abstract

Tsunamis are natural phenomena that, although occasional, can have large impacts on coastal environments and settlements, especially in terms of loss of life. An accurate, detailed and timely assessment of the hazard is essential as input for mitigation strategies both in the long term and during emergencies. This goal is compounded by the high computational cost of simulating an adequate number of scenarios to make robust assessments. To reduce this handicap, alternative methods could be used. Here, an enhanced method for estimating tsunami time series using a one-dimensional convolutional neural network model (1D CNN) is considered. While the use of deep learning for this problem is not new, most of existing research has focused on assessing the capability of a network to reproduce inundation metrics extrema. However, for the context of Tsunami Early Warning, it is equally relevant to assess whether the networks can accurately predict whether inundation would occur or not, and its time series if it does. Hence, a set of 6776 scenarios with magnitudes in the range $M_w$ 8.0–9.2 were used to design several 1D CNN models at two bays that have different hydrodynamic behavior, that would use as input inexpensive low-resolution numerical modeling of tsunami propagation to predict inundation time series at pinpoint locations. In addition, different configuration parameters were also analyzed to outline a methodology for model testing and design, that could be applied elsewhere. The results show that the network models are capable of reproducing inundation time series well, either for small or large flow depths, but also when no inundation was forecast, with minimal instances of false alarms or missed alarms. To further assess the performance, the model was tested with two past tsunamis and compared with actual inundation metrics. The results obtained are promising, and the proposed model could become a reliable alternative for the calculation of tsunami intensity measures in a faster than real time manner. This could complement existing early warning system, by means of an approximate and fast procedure that could allow simulating a larger number of scenarios within the always restricting time frame of tsunami emergencies.

## Introduction

Tsunamis have the potential to cause widespread damage and loss of life, over large swaths of coastal areas. To mitigate their effects, either in the long term or during emergency situations, an accurate and detailed assessment of the hazard is essential. However, this can be affected by two major constraints. The first relates to data accuracy. Under the standard assumption that tsunami hydrodynamics are sufficiently well understood to be described by a mathematical model and its numerical implementation^1–4^, the problem then lies in the accurate determination of the proper initial conditions, i.e., the tsunami source, and the characterization of the boundary conditions such as bathymetry, topography, and its roughness. The second constraint, relevant for tsunami early warning, is that the time allotted to obtain an assessment can be very short, thereby limiting the strategies available to estimate the hazard in minimal times with high accuracy. This contrasts with the need to provide accurate and meaningful information in minimal time to trigger evacuation processes.

Ideally, during an emergency the tsunami hazard assessment would involve an on-demand full forward numerical modeling of the tsunami throughout all its stages (generation, propagation and inundation) using all available data (including the source) to forecast its characteristics before its arrival. However, the short time between tsunami generation and arrival in the near field^5^, has driven the estimates to be based mostly on tsunami propagation modeling. Inundation modeling is computationally expensive owing to the need to use costlier nonlinear models and increased model resolution^6^. Recent advances in very fast tsunami source characterization and modeling using high performance computing may reduce times to make them compatible with Tsunami Early Warning Systems (TEWS) time requirements, either in Near Real Time or even Faster than Real Time^7–12^.

However, epistemic uncertainty limits tsunami source characterization sufficiently to hamper tsunami inundation accuracy^13,14^. Hence, a probabilistic assessment of the hazard might be needed, which require a large number of tsunami modeling runs^13,15–19^, using expensive computer facilities, or extended evaluation times^8,20^. For example, Gusman and Tanioka^20^ report computing times longer than 14 min for a single, site-specific simulation, well in excess of the expected arrival times in places like the eastern Pacific seaboard^5^. One alternative is to keep the focus on propagation modeling to allow for including a larger number of scenarios, but leaving aside inundation modeling^15^. It is noted that the problem of accurate source characterization and the estimation of tsunami hazard are distinct, separate and sequential, although both contribute to the final result. In what follows, the analysis focuses on improving the latter, assuming that the former is available.

Within the context of TEWS, the long time needed to obtain a full forward modeling have prompted the use of strategies that trade off accuracy in favor hazard assessment time. A prime example is the use of databases of precomputed scenarios, as done in TEWS in Japan, Indonesia, Australia and Chile^21–24^. Precomputed databases rely on partial forward modeling (generation and propagation) of predefined tsunami sources of varying rupture lengths, widths and a range of magnitudes, which are queried using simple earthquake data (hypocentral location and magnitude) as its input. This source characterization does not consider uncertainties in predicting actual slip. Rather, these databases rely on an uniform slip distribution, which is known to underestimate peak tsunami intensity metrics such as runup^25,26^. Alternatively, tsunami time series at coastal forecast points can be obtained using a set of unit source functions that are linearly combined to obtain time series of tsunami propagation in coastal waters^27,28^. In either case, the expensive nonlinear modeling of the tsunami is removed from the emergency cycle, and is replaced by faster look up and matching procedures, or linear approximations. Consequently, the inundation stage of the tsunami is usually omitted and the hazard assessment is done over tsunami wave heights as inundation hazard proxies, for instance using Green’s Law and similar approaches^29^.

The apparent requirement to model several scenarios including inundation could overload the computational capacity of most TEWS, prompting Amato^30^ to suggest the need to develop new modeling techniques. One alternative is to use fast-computing of analytical approximations to predict Tsunami Intensity Metrics (henceforth TIMs) such as runup, but these tend to correlate well only close to the source where source effects dominate tsunami hydrodynamics^31–34^. Their accuracy decays rapidly as nonlinear and bathymetric-control processes such as resonance or energy funneling become more dominant during the later stages of the tsunami. This has limited the application of these analytical approaches, prompting again the use of forward modeling and the basic principle of precomputed databases, albeit now aimed to estimate inundation. The less expensive propagation models from uniform slips sources are used to obtain tsunami time series at coastal sites, which become the input for table look-up procedures where tsunami inundation maps become the output^6,23,35–37^. Among these, the NearTIF algorithm^36^ has been evaluated at several locations^38–41^ with good results. However, it requires an inundation database that covers the appropriate parameter space of cases and conditions beforehand.

Finally, it is possible to use emulators, understood as a simpler statistical model that approximates results of a simulator, in this case, the tsunami full forward model. For instance, Gaussian Process have been used to predict maximum free surface displacements using as input minimal data from the tsunami source, such as the earthquake location and magnitude, with reasonable results^7,42^. Another alternative can be the use of Machine Learning techniques such as neural networks, which can be understood as a special type of emulator. These have gained significant attention lately because they can reduce the hazard assessment time significantly, allowing even for the estimation of inundation.

Regarding applications of ML methods to tsunamis, Barman et al.^43^ estimated the tsunami time of arrival (ETA) on a localized region, by training a Multi Layer Perceptron (MLP) network over a larger region, using as input for the network design a database of ETA. Results showed good accuracy with a significant speeding up of computation time. Others have used Artificial Neural Networks (ANNs) to address detection of tsunamis in sensors^44^, or the identification of parameters that control risk rather than hazard^45^. However, regarding tsunami early warning, it is of interest to forecast TIMs such as runup, inundation extent, or flow depths, either as extreme values or time series. Namekar et al.^46^ trained two non-specified neural networks of identical architecture, one to predict free surface time series at coastal points, and the other to predict the runup distribution. Training was performed using a database of synthetic tsunamis from which time series at the location of three Deep ocean Assessment and Reporting of Tsunamis (DART) buoys were used as input, and both coastal time series and runup as outputs. Performance was assessed by comparing against actual data from the 2006 Kuril Island tsunami and its effect on Ohau, Hawai’i, USA, with good results, suggesting the possibility to bypass completely source characterization and use DART data as only input. The opposite philosophy was used by Günaydn and Günaydn^47^, who bypassed tsunami modeling instead, by using both a Feed Forward Back Propagation (FFBP) and a General Regression Neural Network (GRNN) to predict runup based on the focal point data of the earthquake (hypocentral location and moment magnitude), and distance to the point of interest, again with good results. Hadihardaja et al.^48^ also used a GRNN to forecast runup from earthquake source data in Indonesia. It is of note that these latter approaches implicitly assume that runup is controlled by source characteristics, neglecting the contribution of bathymetric controls such as energy funneling and/or trapping, and resonance. Runup forecasting was also tested by Yao et al.^49^ using MLP, although they focused on finding the optimal network configuration. Liu et al.^50^ carried an extensive analysis, on which they combine different neural networks schemes in a sequential manner to address the problem of significant feature extraction, gappy or noisy data, and sparse measurements. The assessment was done for maximum wave amplitude and free surface time series, with good results and providing an assessment of the uncertainty arising from the neural network prediction.

All these works targeted prediction of point statistics of TIMs. An extension of this approach is to obtain their spatial distribution (i.e., maps). For instance, Romano et al.^51^ also use MLP to relate the earthquake source as input data, with maps of maximum tsunami wave height and ETA in coastal waters. Inundation statistics, perhaps owing to its high nonlinearity, have been addressed only recently. Fauzi and Mizutani^52^ used a Convolutional Neural Network (CNN) to optimize the matching algorithm of NearTIF, but also use MLP to directly obtain tsunami inundation maps (i.e., maps of inland maximum tsunami flow depth). The MLP consists of five hidden layers and 128 nodes, to produce prediction based on 328 modeled tsunamis, covering a range of magnitudes although using uniform slip. A Linear Shallow Water Equation (LSWE) model was used to obtain maximum tsunami amplitude offshore on a low resolution grid (30 arcsec) that were paired to tsunami inundation obtained with a NLSWE model at higher resolution (1.11 arcsec). Performance assessment was done using a $M_w$ 8.7 hypothetical event. The relative performance of the models varied significantly, which was associated to both the use of uniform slip for the initial condition which may not be representative enough of the tsunami characteristics and the limited number of scenarios used. Mulia et al.^53^ used a similar approach, considering 532 source scenarios with uniform slip distributions, using also 30 arcsec and 1.11 arcsec resolutions for the modeling. However, a Deep Feed Forward Neural Network using tsunami inundation from a low resolution LSWE model was used as input (instead of coastal tsunami amplitudes), and a high resolution inundation from a NLSWE as model output. They tested the results against data from an observed tsunami, including inversion of the source. Results were found to be very good, both in terms of inundation extent and runup.

These studies used extrema of the variables in the training, thereby discarding their time series. Part of the reason is that time series prediction requires a different neural network architecture. Indeed, Mase et al.^54^ used a FeedForward architecture to predict sea surface elevation inside Osaka Bay, using as input offshore time series at a single location. Mulia et al.^55^ used an Extreme Learning Machine (ELM), to forecast tsunami time series in coastal shallower waters, aiming at reducing the limitations of more common procedure that invokes linear superposition, whereas the ELM includes nonlinear processes. The ELM increase in accuracy was traded off by nearly doubling the computational time, although it was generally less than 0.5 s, thus extremely fast for TEWS applications. Perhaps in the most complete work for TEWS to date, Makinoshima et al.^56^ trained a 1D-CNN to forecast tsunami time series of inundation as generated by a earthquakes in the range of $M_w$ 9.0–9.2, with great accuracy and speed. They use as input data obtained from a dense network of actual tsunameters, as well as geodetic data from GNSS observations. They tested for sensitivity of the neural networks using different configurations of input data. Among the potential downsides of the setup, is the very dense 1D-CNN configuration used, leading to millions of parameters to be determined, and that it requires observational data that might not be available in other parts of the world. In contrast, Liu et al.^50^ used a single location as input data, with short run lengths, to extrapolate time series of free surface elevation. In their case, the nearly one-dimensional flow of the testing site could have facilitated this, although the methodology can be applied elsewhere by including more input data locations.

Hence, it can be seen that Machine Learning techniques offer a promising opportunity to speed up some of the evaluations required for TEWS, especially in terms of inundation, which has been often discarded owing to its large computational burden. However, there are certain aspects that need to be considered further. First, it is worth assessing whether the neural network model can reproduce not only cases of tsunami inundation, but also cases of no inundation with equal success, to reduce the possibility of hazard over estimation (false alarms) or underestimation (not triggering an alarm), something that has not been addressed. This requires testing for a large number of scenarios. A good neural network model should be capable to predict not only the flow depth but also its temporal features, such as arrival time and time of the peak. Second, it is needed to assess what would be the minimal requirements of a neural network design that still offers good solutions. Dense neural networks with large amounts of actual input data such as the one used by Makinoshima et al.^56^ might not be currently feasible elsewhere.

The present work aims to addressing these questions while providing criteria for defining the training and testing data sets. To this end, a similar configuration to that of Makinoshima et al.^56^ is evaluated, but considering three main differences: (i) similar to prior research, a low resolution forecast obtained from the numerical modeling of tsunami propagation is considered to be the input data^52,53^. The reason for this is to assess how capable is the network model to forecast in cases where offshore sea surface time series are not readily available or suitable to be used by a neural network, as occurs in many countries; (ii) A wider range of scenarios are tested. While most of the prior work has focused on the determination of the capability of a network to reproduce inundation values, for the context of TEWS it is equally relevant to assess whether the networks can predict tsunami occurrence with no inundation, to minimize false alarms and (iii) A neural network with fewer parameters is trained, aiming to simplify the training process.

It is expected that a simple and accurate network model can aid in operational TEWS in the sense that, by reducing the time required to compute inundation, it can allow modeling a larger number of scenarios, for instance, to account for uncertainty in source characterization. The simple model evaluated herein, can be further expanded to incorporate additional features such as those proposed by Liu et al.^50^.

## Data and methods

### Tsunami data

For the present implementation, the inundation resulting for a range of tsunamigenic earthquakes at two locations in central Chile are considered. First, the highly exposed area of the cities of Valparaíso and Viña del Mar (33$^\circ$01’28”S 71$^\circ$33’06”W). This region has not been significantly affected by tsunamis generated by the most recent local earthquakes such as those of 1906 or 1985^57^; nor by regional tsunamis such as Maule 2010^58^, Pisagua 2014^59^, and Illapel 2015^60^; nor the far field 2011 Tohoku transpacific tsunami. However, a large local earthquake in 1730 did inundate the floodplain in Valparaíso^57^. This varying behavior makes it a suitable location for studying the forecasting capabilities of a neural network model aimed at determining the occurrence of inundation for a wide range of earthquake magnitudes. The other location is Coquimbo bay (29$^\circ$57’12”S 71$^\circ$20’17”W), which shows a different behavior, as it was inundated during the 2015 Illapel earthquake^60^, while large amplitudes were recorded in the local tide gauge during the 2010 Maule tsunami^58^ and recently during the Hunga-Tonga-Hunga-Ha’apai transpacific tsunami^61^, but without inundation. In addition, the bay is highly resonant, and with a spatial structure of the first mode that induces the southern end of the bay to be susceptible to tsunami inundation, whereas the central and northern ends are less prone to become inundated^62^. Hence, this location allows for assessing the capabilities of the network for complex inundation behavior and inundation footprints.

**Figure 1:**
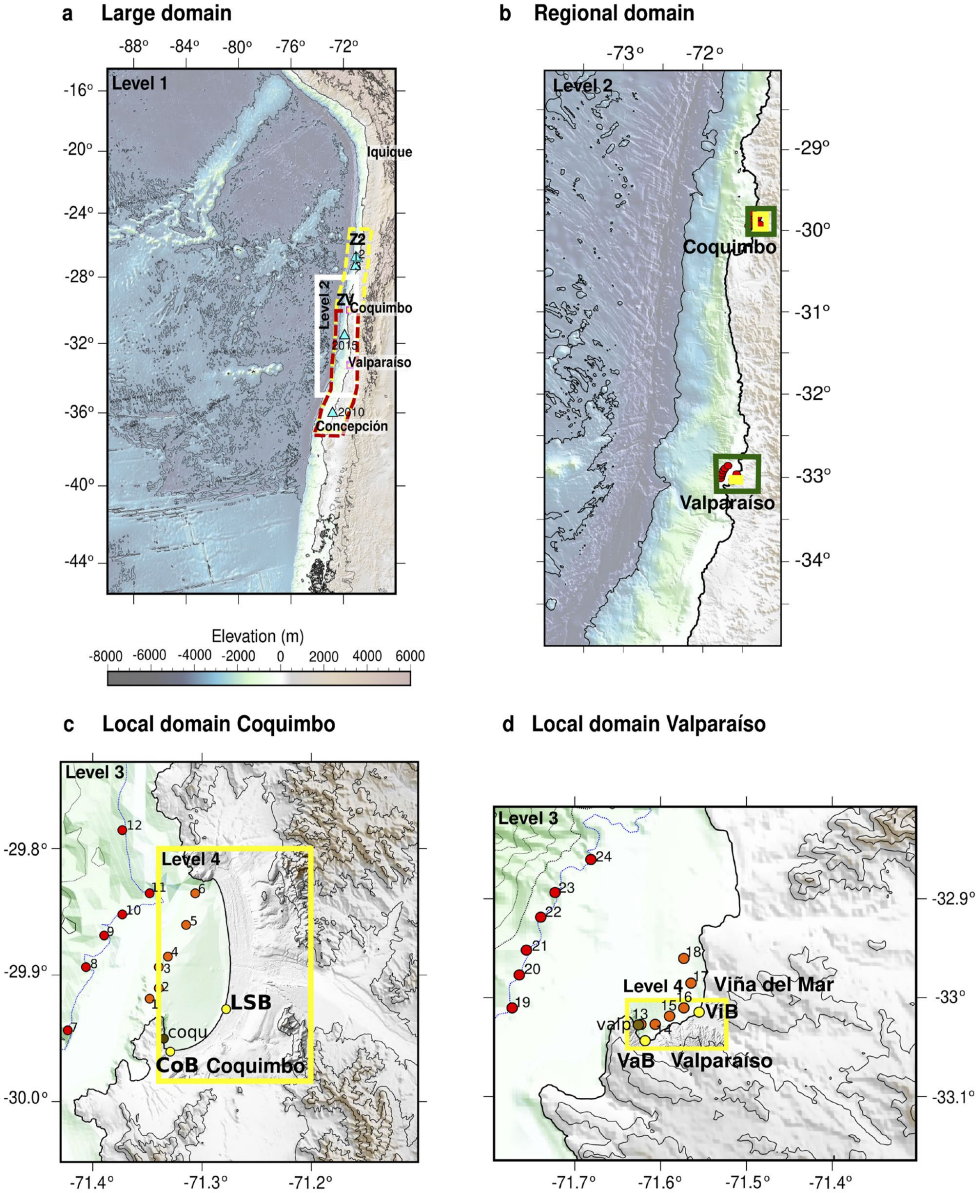
Topo-bathymetry and nested domains used for tsunami numerical simulations with Tsunami-HySEA. (**a**) Basin domain (Level 1) at spatial resolution of 1 arcmin. Yellow dashed box shows Zone 2 of Poulos et al.^63^ and ZV the study area. The 2010 and 2015 (cyan triangles) correspond to the location of centroids taken from the Global Centroid Moment Tensor database. White box denotes Level 2. (**b**) Regional (Level 2) grid with resolution of 15 arcsec. Black boxes indicate the two local domains. c and d) Local (Level 3) grid with resolution of 1.875 arcsec. Red and orange dots indicate the location of offshore virtual buoys used to obtain input time series, located at the isobaths of 200 m and 50 m respectively. Yellow dots show the location of inland numerical buoys that are the target of the network. Yellow boxes denote the high resolution grid (Level 4, 0.234 arcsec, $\sim 7$ m). Panels c and d correspond to Coquimbo-La Serena and Valparaíso-Viña del Mar, respectively.

The inundation characteristics at both bays were estimated using the numerical NLSWE model Tsunami-HySEA, which has been benchmarked and validated in accordance with U.S. National Tsunami Hazard Mitigation Program (NTHMP)^10,64^. Four sets of nested grids, with spatial resolutions of 30, 15, 1.875 y 0.234 arcsec, were built from the freely available General Bathymetric Chart of the Oceans^65^, and Nautical Charts elaborated by the Hydrographic and Oceanographic Service of the Chilean Navy (SHOA) (Fig. 1). Two types of TIMs were recorded from these model runs. First, two independent sets of numerical coastal buoys located at depths of 200 m and 50 m, were used to obtain free surface time series, $\eta ^{LR}_\ell (t)$, $\ell =1\ldots F$, with $F=6$ coastal buoys per set (shown as red and orange circles in Fig. 1, respectively). These low resolution (*LR*) series will be treated as input data, and their spatial arrangement aims at capturing tsunamis coming from different directions relative to the area of interest. These data were collected from simulations using only the coarsest grid at 30 arcsec, with the objective to train the network to be fed with fast, linear simulations of propagation during an emergency.

Second, high resolution (*HR*) time series of tsunami inundation flow depths $d^{HR}(t)$ were modeled at a set of pin-point locations along the shorelines of either bay (shown as yellow dots in Fig. 1). This marks a departure from previous studies in the sense that rather than estimating the overall inundation map, the aim here is to capture the characteristics of tsunami inundation at specific locations. The underlying hypothesis is that tsunami hydrodynamics may differ even between closely spaced points owing to processes such as resonance, and the designed neural network models (henceforth NNM) for each location can resolve these local features at less cost than tsunami maps. While this may be considered to limit the extent of application of the methodology, the approach followed here can be applied to more points, even further inland, without loss of generality, as proposed by Liu et al.^50^. For the present implementation, these target time series are obtained from tsunami modeling simulations using the highest resolution as proxy for actual inundation patterns. Thus, no real tsunami data are considered. Consequently, this exercise aims to reduce the time of the hazard assessment, provided a source characterization is available by other means.

As a result of this arrangement, six common offshore buoys modeled with the coarse 30 arcsec domain are used for each local domain (Valparaíso and Viña del Mar, Coquimbo and La Serena), and one inland gauge for each city, at high spatial resolution. These were located close to the shoreline, at relative low elevation (see Table 1) . In what follows, these inland gauges are denoted VaB, ViB, CoB and LSB (the first couple of letters refer to the city of interest). Therefore, four NNMs were trained independently. All time series were sampled at 10 sec with a tsunami duration of 6 hours, using a standard Manning roughness coefficient of $n=0.025$ m$^{-1/3}$s.

The initial conditions for the tsunami simulations were estimated from subduction earthquakes taking place along the extent of the $M_w$ $9.1-9.3$ Valparaíso earthquake^57^, partly within the so-called Zone 2 of the zonification proposed by Poulos et al.^63^, respectively shown as ZV and Z2 in (Fig. 1a). A set of 6776 earthquakes with magnitudes in the range $M_w$ 8.0 to 9.2, with 0.1 $M_w$ increments were used. The reason for this range is the existing record of locally generated tsunamis in the region, that comprise $M_w$ 7.8 (1985^66^), $\approx 8.0$ (1906^67^), $8.1-8.4$ (1922^67^ and 2015^60^), and the estimated $9.1-9.3$ (1730^57^). Among these, 1730 was the only one to have inundation in Valparaíso, whereas 1922 and 2015 did cause inundation in Coquimbo. However, Zamora et al.^68^ found that events $M_w$ 9.0 are also capable of inundating the Valparaíso region, depending on the characteristics of the slip distribution. Hence, to account for source variability, these synthetic earthquakes were generated within this region using the Karhoenen-Loeve Expansion following Leveque et al.^69^ and Melgar et al.^70^, considering a domain discretization of 10 x 10 km. Details on the characterization of the source data are available in the Supplemental Material.

In addition, for assessing the performance with completely unknown data, different rupture models were used as input conditions for two historical events. For Maule 2010, these consider the best-performing median model reviewed by Cienfuegos et al.^13^, and the sources estimated by Hayes (NEIC)^71^ and Benavente and Cummins^72^ were arbitrarily selected. Similarly for Illapel 2015, the sources from Okuwaki et al.^73^, Shrivastava et al.^74^ and the solution from Hayes^75^ (and also available in the webpage of the event, mantained by the United States Geological Survey) are chosen only as reference for this study. Most of these sources are available at SRCMOD^71^.

All slip models were transformed to free surface elevation using the Okada^76^ solution for surface displacement considering tapering and a Kajiura filter, before running each simulation. Simulations were run on the CTE-Power9 system at the Barcelona Super Computing Center servers using four Graphic Processing Units (GPUs) and 40 Central Processing Units (CPUs) per task. The entire high resolution 6776 scenarios dataset was generated, on average, in 400 hours of computational time, and the low resolution runs took about 35 hours. The CTE-Power9 system has two login nodes and 52 computing nodes. Each of them accounts for two IBM Power9 processors (20 cores, 160 HT) and four NVIDIA Volta V100 GPUs.

### Database pre-processing

**Figure 2:**
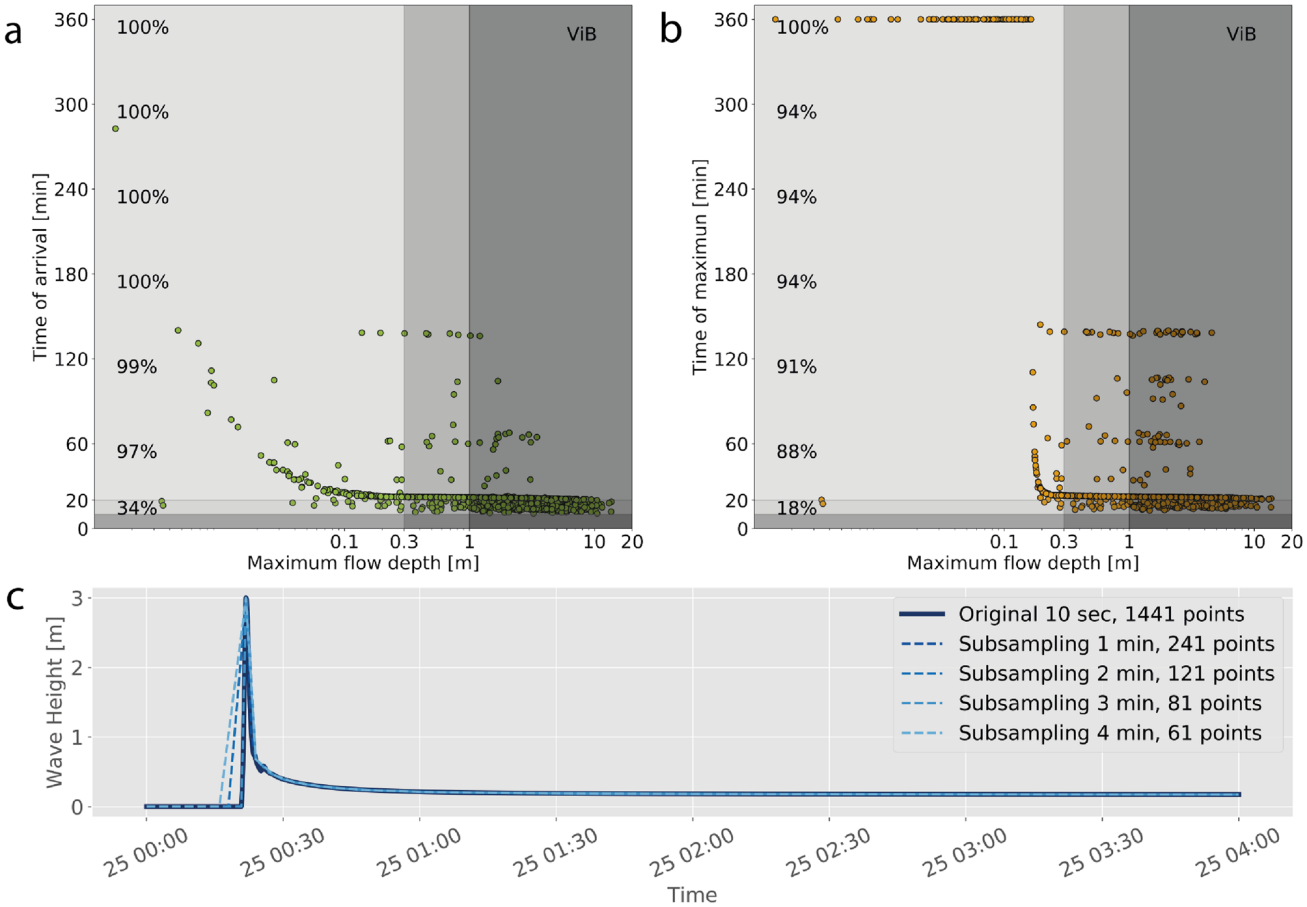
Scatter plots of the joint distribution of (**a**) maximum flow depth and tsunami arrival time; (**b**) maximum flow depth and time of the maximum, for all scenarios (dots). Vertical colored regions distinguish between the tsunami amplitude values used to categorize the hazard^21^. Horizontal coloring distinguish between very fast, fast and late arrivals, following^68^. Percentage values on the left indicate the cumulative fraction of events as a function of time. (**c**) Sample time series of inundation, showing the effect of different sub-sampling values.

The amount of data contained on each of the input and target datasets has a direct relationship with the number of network parameters to be trained, thereby requiring the use of dense and deep networks if a high level of detail is required. The present implementation aims to assess whether networks with fewer parameters can succeed in providing meaningful early warnings, discriminating between inundation or no inundation. Hence, some data pre-processing was performed based on two criteria. First, to determine the minimum length of the time series that carry relevant information for the assessment. On this regard, even though six hours of tsunami records were modeled, the meaningful parameters are the time when first arrival occurred (denoting that inundation has taken place), and the time of maximum flow depth (assumed to be the worst condition). At each inland gauge, the joint distribution of maximum flow depth versus arrival time, and time of peak amplitude were estimated. A sample of these distributions is shown in Fig. 2 for ViB. The tsunami arrival time is recorded internally by Tsunami-HySEA as the first instance of non-zero flow depth inland, and most of the arrivals ($97\%$) occur within an hour (see the percentage index on the left of the graph in Fig. 2a), while the flow depth of the first varying significantly in magnitude. On the other hand, the time of peak flow depth was calculated independently and is shown in Fig. 2b. There is no one-to-one correlation between the two metrics, since many of the peak flow depths take place within 3 hours after tsunami onset. A small number of very low magnitude flow depths occur very late in the simulation. From this analysis, it is possible to conclude that the most meaningful information can be obtained even if the time series length is trimmed. To provide an objective measure for this, it was defined that the run length had to satisfy that 99% of the cases have arrived, and that at least 90% of the peaks have been included. This allowed to reduce the time series for the network training to four hours in Coquimbo-La Serena, and two hours for Valparaíso-Viña del Mar. Here, differences in hydrodynamics appear to play a role.

The second consideration that affects the number of parameters is the resolution of the time series. While the model runs were designed to provide outputs every 10 s, this could be an excess of information for tsunamis which are long period waves in the range of several minutes. Hence, it is possible to subsample the series in such a way that relevant features of the time series are retained. However, unlike time series in water, inundation can have short lived features due to the episodic and complex nature of inundation flows. Hence, a sensitivity analysis for subsampling was carried out as shown in Fig. 2c, where a high resolution time series (solid dark line) is compared with subsampled ones. Subsampling at 1 min (60 s) suffices to retain key features such as the timing of first arrival, secondary peaks and late arrivals. This allows reducing the total number of samples to 1/6-th of the original size. The combined effect of trimming and subsampling led to time series with a total number of samples $\alpha =$ 241 and 121 for Coquimbo-La Serena, and Valparaíso-Viña del Mar, respectively, down from $\alpha$=1441 and 721 data points of the length-trimmed 10 sec series. In addition, the NNMs can produce noisy time series that can affect the comparison against no inundation cases, without affecting the hazard assessment. Consequently, all NNM-predicted flow depth values less than 5 cm were treated as zero. For completeness, the analysis will be carried out to compare network performance with both sampling rates to assess the impact of this on the performance of the networks.

**Table 1:** Distribution of scenarios that do and do not inundate at each inland numerical sensor.

Buoy	Zone	Elev.	Do	Do Not
		[m] above	Inundate	Inundate
Id		MSL	[%]	[%]
CoB	Coquimbo	1.96	47.0	53.0
LSB	La Serena	2.69	25.2	74.8
VaB	Valparaíso	4.56	11.2	88.8
ViB	Viña del Mar	4.77	20.5	79.5

A final data processing relevant for machine learning training, is to provide training data sets that show class balancing. This is understood as that the data sets ought to contain comparable number of cases for each of the categories that are to be discriminated. In the present case, the wide range of magnitudes used could induce that a disproportionate number of scenarios may not induce inundation. On the other hand, considering only large scenarios can imbalance the data set towards inundation. Table 1 shows the overall ratio of scenarios that inundate each buoy over the entire 6776 data sets. It can be noticed that the Coquimbo inland gauge CoB shows a larger tendency to be inundated, with 47% of the cases triggering inundation. La Serena, despite being close to Coquimbo, gets inundated only 25% of the time, which highlights the relevance of distinguishing between the hydrodynamics of neighboring points. Viña del Mar is twice as susceptible than Valparaíso, that gets inundated a mere 10% of the cases. This wide range of results poses a challenge for class balancing, as locations as Valparaíso might lead to over representing no inundation cases. To account for this, for each location of interest, the data sets of training, testing and validation were designed to retain each gauges’s overall percentage, without paying attention to other discrimination criteria.

### Network architecture

Machine learning techniques have gained significant attention over the last few years and multiple applications. Among these, sequence to sequence (Seq2Seq) aims at developing models that convert sequences in one domain, to corresponding sequences in another domain. In the present implementation, the goal is to find a network that can convert sequences (in this case time series) of simulated free surface elevation in coastal waters $\eta ^{LR}(t)$, to sequences of flow depth $d^{HR}(t)$ on inland terrain. These are treated as different domains, as the former are usually continuous series of real values (negative and positive), whereas the latter can be discontinuous occurrences of positive only values, if any.

Earlier Seq2Seq network architectures designed for signal processing, such as FeedForward or Multi Layer Perceptron (MLP)^77^, assume independence among variables. Hence, the presence of temporal or spatial dependencies degraded its performance^78,79^. To overcome this, recurrent links allow for transfer of information among different time steps^80^. However, these early Recurrent Neural Network architectures were computationally expensive, and were subject to instabilities associated with fading and/or large gradients when long term processes were present^81^. Hochreiter and Schmidhuber^82^ proposed the Long Short-Term Memory (LSTM) model aimed to reduce fading for long term dependencies. Despite these advances in sequence to sequence models, the success of Convolutional Neural Networks (CNN) in identifying complex patterns and objects in image and video processing, has prompted its use in signal processing with good results^83^. Among these, 1D CNN^84^ and their compact implementations show good results when data are limited. Moreover, they do not require high-end hardware and a single CPU can suffice for training^83^.

Both Seq2Seq and CNN architectures have been applied to tsunami inundation. Fauzi and Mizutami^52^ used a CNN to classify low resolution tsunami inundation maps, and MLP to model and map these low resolution series to the inundation map. Mulia et al.^53^ expanded on this, by incorporating a larger number of scenarios to calibrate a Feed Forward model with several hidden layers, aimed to characterize more complex attributes. Both works focus on inundation maps, whereas Makinoshima et al.^56^ use a deep 1D CNN to estimate tsunami inundation time series based on the input series obtained from a dense network of tsunameters deployed in Japan, as well as geodetic information. They used 49 offshore observation points coincident with actual bottom pressure sensor locations, and five geodetic points from the Global Navigation Satellite Systems network. The network was trained with 12,000 stochastic scenarios generated within the rupture domain of the 2011 Tohoku Earthquake. The network offered good performance under varied combinations of input data and observation times. For the case of predicting time series, Liu et al.^50^ also used a CNN coupled with a Denoising Auto Encoder (DAE) and a Variational Auto Decoder. An encoder (DAE) is used to denoise and correct noisy or gappy input data, whereas the decoder (VAD) estimates confidence bounds on the prediction of time series in coastal waters. While they did use sparse data, it could have been alleviated by the relatively simple configuration of their problem setting, that resembled a one dimensional channel. This is a difference from prior machine learning studies regarding tsunamis, that have focused on predicting time series in coastal waters or inundation TIMs maxima. Only Makinoshima et al.^56^ used time series of inundation, but with large magnitude events.

**Table 2:** Space of hyper-parameters tested.

Hyperparameters	Range of values	
Variable	60 s	10 s
Kernel size $\beta$	[**9**; 12]	[18; **27**]
Kernel stride $\gamma$	[**3**; 4]	[15; **21**]
Neurons D1	[L/4; **L/2**; L; 2L]	[L/4; **L/2**; L; 2L]
Neurons D2	[L/4; L/2; **L**; 2L]	[L/4;** L/2**; L; 2L]
Buoy Depth (m)	[**50**; 200]	[50; **200**]
**Constant**		
Dense layers	2	
Convolutional layers	3	
Patience	10	
Dense layers activation function	Linear	
Convolutional layers activation function	ReLu	
Filters	64-32-16	
Dropout *p*%	10.0	
Batch size	32	
Learning rate	${10}^{-3}$	
Output layer activation function	ReLu	

Here, compact 1D CNN network architectures are designed, determining both their hyper-parameters and parameters, with the main focus of estimating the predictive ability for inland inundation. The methodology is implemented in Python 3.6, using the Keras API within TensorFlow, with an ADAM optimizer with a learning rate of 0.001, on a consumer-level portable computer.

In CNN terminology, “hyper-parameters” are understood as user-defined parameters that constrain the network architecture and its performance, whereas the term “parameters” refers to the actual weights of the neurons that optimize the network predictive ability. An initial set of 16 hyper-parameters was considered. However, upon early examination and sensitivity analyses, ten of these were fixed thereby leaving only six to be determined. These are shown in Table 2, where hyper-parameters that are being selected through validation are shown in bold. Reducing the number of hyper-parameters allows for reducing the computational training time. Although they are not hyper-parameters in the strict sense, the analysis also includes two experimental design variables. First, the use of time series with the original and reduced sampling rates (columns labeled 60 and 10 s in Table 2). Second, the choice of input time series located at either 200 or 50 m water depths (hyper-parameter Buoy Depth). *L* represents the length, in samples, of the target time series $d^{HR(t)}$ at inland gauges.

**Figure 3:**
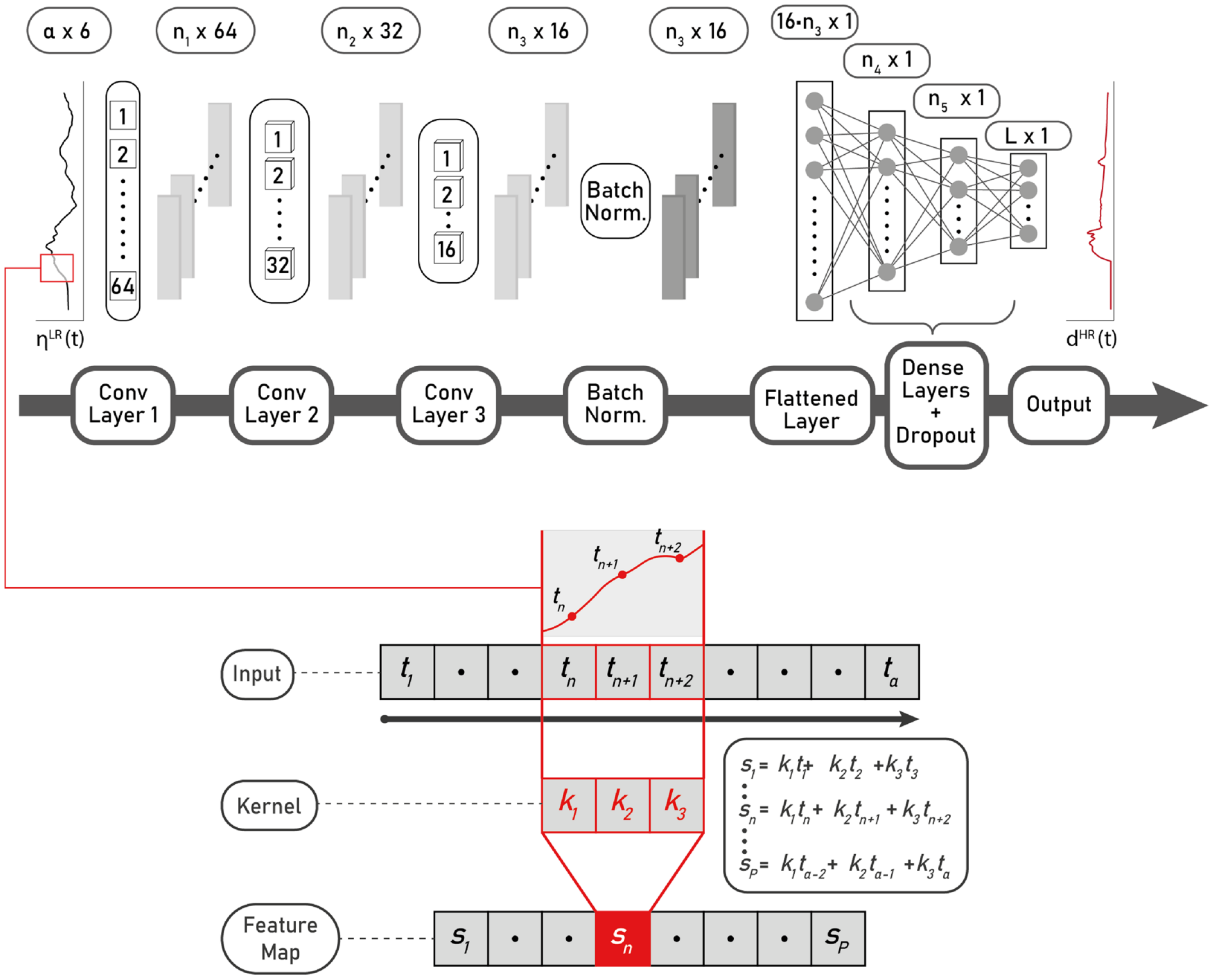
Schematic of the network architecture. Top panel shows all steps using the nomenclature specified in the text. Bottom panel shows represents the feature extraction from a convolutional layer.

A schematic of the 1D CNN architecture is shown in Fig. 3. The leftmost panel represents one of the six buoy time series $\eta ^{LR}(t)$ that are located in offshore waters, at one of the tested depths. Each of these time series is represented as a sequence, $\mathbf {\eta _{\ell }} : \{t_1^{(\ell )},t_2^{(\ell )}, \dots , t_\alpha ^{(\ell )}\} \rightarrow {\mathbb {R}}^{\alpha }$ with $\alpha \in {\mathbb {N}}$ the number of input samples, and $\ell$ indexes the $F=6$ different coastal buoy time series. All of these are fed into the first convolutional layer, where a kernel is applied. A kernel is a vector of fixed weights $\mathbf{k} ^{(\ell )} : \{k_{1}^{(\ell )},k_{2}^{(\ell )},\ldots ,k_{\beta }^{(\ell )}\} \rightarrow {\mathbb {R}}^{\beta }$ of length $\beta \in {\mathbb {N}}$ (a hyper-parameter) and where $\ell$ indexes the different dimensions of the kernel, which must be equal to the number of input dimensions of *F*. This kernel is applied as an inner product, sweeping sequentially the series to obtain a feature map. The kernel covers the entire input sequence using a staggered stepping size (hyper-parameter kernel stride = $\gamma$), hence the convolution. Formally, a neuron attribute (also called a feature in CNN terminology) is computed as

1 $$\begin{aligned} s_{n} = (\mathbf {\eta } *\mathbf{k} )(n)=\sum _{\ell = 1}^F\left( \sum _{p=-\infty }^{\infty }{\eta ^{(\ell )}[p]\cdot k^{(\ell )}[p-n+1]}\right) , \end{aligned}$$

where $n \in \{1,\ldots , \lfloor \frac{\alpha - \beta }{\gamma } \rfloor +1\}$ and by convention when a sequence is evaluated out of its domain of definition the result is zero, thus not contributing to the sum. This sequence defines the feature map $\mathbf{s}$ associated to the kernel $\mathbf{k}$. Padding the series was not considered.

A convolutional layer is defined by several kernel configurations, also denoted as filters. The input is processed simultaneously by all kernels, obtaining a sequence for each kernel. Thus the output of a convolutional layer is a filtered multidimensional sequence. Next, a non-linear activation function $\sigma$ is applied to this sequence, obtaining the output *y* of the convolutional layer:

2 $$\begin{aligned} y(n) = \sigma \left( b +s_n\right) , \end{aligned}$$

where $b \in {\mathbb {R}}$ is an intercept, one of the network trainable parameters, and $\sigma : {\mathbb {R}} \rightarrow {\mathbb {R}}$. Here, a rectified lineal function^85^ is used through the network, defined as ReLu$(x) = \max \{x,0\}$.

Following this, the resulting sequence *y*(*n*) is fed into the following convolutional layer, where the process is repeated with different kernels. The consecutive application of the process highlights relevant features in the series. The total number of convolutional layers can be treated as a hyper-parameter, but here only three layers were considered. Moreover, the number of filters in each convolutional layer is also treated as a constant (cf. Table 2).

After the process of convolution, a batch normalization is applied^86^, aimed to minimize the risk of generating values drastically different to the learned distribution, and propagating errors down the layers. The resulting flattened layer, is then fed into two dense layers. These follow the scheme of fully connected layers, similar to MLP, where all the attributes $\mathbf{a} ^{(l)}$ of a previous layer *l*, are subject to a vector of weights $\mathbf{w} _{u}^{(l)}: \{{w_{1u}^{(l)}, w_{2u}^{(l)},\ldots ,w_{Iu}^{(l)}}\} \in {\mathbb {R}}^{I}$. Hence, the output of the attribute *u* of the layer ${l+1}$ is defined as

3 $$\begin{aligned} \begin{aligned} a_{u}^{(l+1)}&= \sigma \left( \mathbf{w} _{u}^{(l)T}{} \mathbf{a} ^{(l)} + b_{u}^{(l)}\right) \\&= \sigma \left( \sum _{i=1}^{I}w_{ui}^{(l)}a_{i}^{(l)} + w_{u0}^{(l)}\right) \end{aligned} \end{aligned}$$

To reduce the risk of overfitting, a Dropout^87^ layer is applied after each dense layer, where a fraction *p* of neurons are randomly discarded during training. Finally, the length of the dense layers was also considered as a hyper-parameter, defined as different fractions of the total number of samples *L* in the target time series. While in general applications $\alpha$ and *L* are not required to be identical, in the present implementation the number of samples in the input and target series are equal, i.e. $\alpha =L$.

After the last layer, the ReLu activation function is used again. The rationale behind this is that the inundation time series can take values equal to zero (no inundation) or positive when inundation occurs, and that the shape of the series is relevant. ReLu allows for positive values only and hence appears to be best suited for this task.

### Validation and training data sets

**Figure 4:**
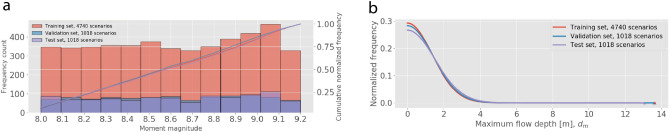
Statistics of the distribution of scenarios among validation, training and test data sets for ViB. (**a**) Distribution as function of moment magnitude (bars) and normalized cumulative frequency. (**b**) Frequency distribution in terms of maximum flow depth $max\{d^{HR}\}$. Symbols denote the extrema of each set. Additional plots for other inland gauges can be found in Supplemental Material.

The objective is to design a NNM at each of the four target locations following a multiple step process. First, validation, understood as the process aimed to determine the hyper-parameters of the NNM. Next, training, where the parameters (weights and intercepts) of the hyper-combination of choice are determined. Finally, testing involves assessing the best model performance for scenarios the NNM has not seen before. In this case, the total of 6776 scenarios are divided in two independent sets of about 1017 scenarios each (15%) used for validation and testing, and a third set of 4742 (70%) for training. Selection of the scenarios was done by randomly selecting scenarios, but retaining the relative percentages of inundating and non inundating scenarios (cf. Table 1). The procedure is done independently at each of the four target locations. In Fig. 4a, the histograms of maximum flow depth of the testing, training and validation sets are shown for ViB (plots for the remainder inland gauges are shown in the Fig. S6 of the Supplemental Material). The distribution of maximum flow depths are similar among data sets, with slight differences in their extrema (symbols). However, these differences are well in excess of the maximum hazard threshold and the training set has the largest value, thereby during training the worst condition is considered. Figure 4b shows the distribution of the scenarios in terms of magnitude, both in bars and as a cumulative function (lines). The three data sets show a similar distribution, hence the scenario space is well distributed among sets.

Owing to the large number of hyper-parameters, an exploratory assessment was done only in Viña del Mar to determine the hyper-parameters that were treated as constant. Upon this selection, 256 combinations of the remainder hyper-parameters were run with the validation data set. The end result are NNMs that take the input sequences $\eta ^{LR}_{(\ell )}(t)\approx \eta _\ell =t_1,t_2, \dots , t_\alpha ,$ aimed to map $F_j(t_1,t_2, \dots , t_\alpha ) \rightarrow Y_1, Y_2, \dots , Y_L$. $F_j$ represents the *j*-th NNM (a combination of hyper-parameters and parameters), and $Y_i$ the time series $d^{HR}(t)$ at each inland gauge. To select the NNM that yields the best overall performance, the Mean Squared Error is estimated

4 $$\begin{aligned} MSE_j = \frac{1}{N_S}\sum _{i = 1}^{N_S}\sum _{k=1}^{L}\left( F_{j,i}(\eta _{\ell })_k-Y_{i,k}\right) ^{2}, \end{aligned}$$

where $N_S$ is the number of scenarios used in the analysis. However, the MSE statistics can be subject to bias due to a large number of small values, especially for the case of no inundation. As an additional metric, a normalized least-squares is also estimated as^88,89^

5 $$\begin{aligned} G_j=\frac{1}{N_S}\sum _{i = 1}^{N_S} \left[ 1-2\frac{\sum _{k=1}^{L}F_{j,i}(\eta _{\ell })_k*Y_{i,k}}{\sum _{k=1}^{L}F_{j,i}^2(\eta _{\ell })_k+\sum _{k=1}^{L}Y^2_{i,k})}\right] , \end{aligned}$$

where the formulation without weights has been used^89^. $G_j$ ranges [0,1], with lower values indicating better accuracy. However, for the case of no inundation, $Y_i=0$ at all times, leading to $G_j$=1 regardless of the value of $F_{j,i}(\eta _{\ell })$, thereby biasing the estimate. Hence, a filter was imposed, such that if the maximum value $max\{F_{j,i}(\eta _{\ell })\}\le 0.05$ m, then $G_j=0$, thus representing perfect agreement for non inundating cases. The 5 cm threshold is arbitrary but small enough not to affect results significantly. The filter was applied a total of 613 times over an accumulate of 3067 non-inundating cases among all four inland gauges, representing a 20% .

Each of these 256 combinations were repeated using five different seeds, and the average value of $MSE_j, G_j$ among seeds was used as the metric of comparison. For each target inland gauge, the optimal hyper-parameter combination was used in training, from which the resulting NNM were obtained. These were then evaluated in testing and with the historical events.

Even though the procedure above leads to a NNM that minimizes the error among the modeled and target time series, it is relevant to assess performance with metrics that are relevant for a TEWS. Especially, whether the peak inundation flow depth, the arrival time, or time of the peak are reproduced satisfactorily. Each of these quantities are assessed by means of the error between observed data and model predictions. In the case of the maximum flow depth, the comparison is between the maximum flow depth in the Tsunami-HySEA series, and the predicted value by the NNM at the same time in the series. This is akin to assess how well the timing and the value of the maximum flow depth are predicted

6 $$\begin{aligned} E_{d,i}= F_{i}(t=t_{max\{Y_i\}})-max\{Y_i\}, \end{aligned}$$

and the difference in arrival time, $t^a$

7 $$\begin{aligned} E_{t,i}= t^a_{F_i}-t^a_{\{Y_i\}}. \end{aligned}$$

Note the index *j* has been dropped because these are the final NNM evaluated at each $i-$th scenario.

However, it is known that TEWS can be less susceptible to absolute errors in these quantities as long as the hazard is categorized properly. That means that, even though an error of 50 cm in peak flow depth might be considered significant, it is not necessarily relevant if both values lead to the same hazard category. Consequently, it is also evaluated whether hazard predictions are consistent between the NNM and data. The focus is set in two relevant cases: (i) whether the NNM overpredicts the hazard (a false alarm), or (ii) whether the NNM underpredicts the hazard (a missed alarm). Both are equally relevant for a TEWS although, arguably, the latter can have more serious consequences. The evaluation is based on the total number of instances where the NNM prediction falls into either category, from the total data set. These results are classified depending on the hazard assessment used in the Chilean TEWS^21^. It is noted that this hazard assessment was devised using as TIM the peak coastal amplitude (PCA), but here the values are retained for reference, as no TEWS uses inundation metrics to date. The most hazardous category is denoted Category C, when the flow depth $d_{max}>$ 1.0 m, prompting full evacuation. Category B is when 0.30 m $< d_{max} \le$ 1.0 m, prompting evacuation of beaches, and Category A is when $d_{max} \le$ 0.30 m, when no action is necessary.

**Table 3:** Ranking of the best performing hyper-parameter combinations in validation.

Hyperparameters						*MSE*	
						Eq. (4)	
Sampl.	Prof.	Neurons	Neurons	Kern.	Kern.	Validation	Training
Rate, s	m	Layer 1	Layer 2	size	stride		
10	200	L/4	L/4	27	15	0.01270	0.00792
10	200	L	L/2	27	21	0.01279	0.00808
10	50	L/4	L/2	18	21	0.01280	0.009269
**10**	**200**	**L/2**	**L/2**	**27**	**21**	**0.01290**	**0.00758**
10	200	L/4	L/2	18	21	0.01301	0.008312
60	50	L/2	L	12	3	0.01479	0.00941
**60**	**50**	**L/2**	**L**	**9**	**3**	**0.01504**	**0.00891**
60	50	L/2	L/2	9	4	0.01478	0.00979
60	50	L	L/2	9	3	0.01487	0.01090
60	50	L	L/4	9	4	0.01496	0.00836

**Table 4:** Perfomance metrics for the best NNM at each location and sampling rate. Data highlighted in gray are the final NNM of each target buoy.

Network	*MSE* Val.		*G* Val.		Validation		False Alarm			Missed Alarm		
	Eq. (4)		Eq. (5)									
	Inund.	No Inund.	Inund.	No Inund.	$E_{d,i}$	$E_{t,i}$						
Model	$[m^{2}]$	$[m^{2}]$	$[-]$	$[-]$	[*m*]	[*min*]	A	B	C	A	B	C
CoB-200-10	0.0159	2.79 E-05	0.1817	0.0112	0.35	12.54	6	0	0	0	0	0
**CoB-50-60**	0.0179	1.96 E-06	0.2154	0.0056	0.33	8.03	3	0	0	12	0	0
LSB-200-10	0.0108	1.63E-06	0.2449	0.0026	0.20	8.4	2	0	0	29	1	0
**LSB-50-60**	0.0111	1.84E-06	0.2381	0.0039	0.25	9.36	3	0	0	12	1	0
ViB-200-10	0.0346	1.75E-04	0.1021	0.1131	0.36	3.19	90	3	0	0	0	0
**ViB-50-60**	0.0226	4.99E-05	0.0908	0.0207	0.20	2.05	16	1	0	3	0	0
VaB-200-10	0.0595	4.95E-05	0.2547	0.0175	0.63	5.68	15	1	0	7	1	0
**VaB-50-60**	0.0337	6.17E-05	0.2382	0.0087	0.37	5.46	7	1	0	7	2	0

The overarching goal of this work is to assess the applicability of a machine learning implementation within the context of a TEWS, especially regarding the capability to distinguish between situations that do inundate from those that do not.Therefore, a final assessment step is to compare NMM predictions against historical data. In particular, data from the recent Maule 2010 and Illapel 2015 earthquakes and their tsunamis can be used for NMM prediction, and the results compared with actual outcomes. Fritz et al.^58^, Aránguiz et al.^60^ and Contreras-López et al.^90^ provide actual inundation data close to the inland gauges of interest, for a reasonable comparison. During both events, ViB and VaB did not suffer inundation, whereas CoB and LSB were inundated only in 2015.

The sources of the two historical events were simulated using Tsunami-HySEA using only the coarsest grid, and the low resolution time series of free surface elevation were obtained at the location of the six offshore buoys, $\eta ^{LR}_{(\ell )}$. These time series were then passed on to each of the previously obtained NNMs, to estimate whether inundation would occur or not, and to categorize it. To better understand possible sources of error, tsunami modeling using the high-resolution nested grids was also performed. This allows contrasting between the simplified hazard assessment flow using the 1D CNN models, and a high resolution modeling similar to what can be expected in Near Real Time modeling.

## Results

The procedure described above was applied to find eight NNM: one for each of the four inland gauges, with a choice of two depths for the offshore buoys (50 and 200 m). The use of two offshore buoy depths was considered to evaluate whether the assumption of wave linearity is relevant for model performance. For each of these NNM, each hyper-parameter combination yields a *MSE*, *G* pair. From these, the top five giving the best performance (minimum *MSE*) were initially selected using a grid search. These are presented in Table 3 for CoB, for reference, classified by the sampling rate. Typical *MSE* values fluctuate in the range 0.01-0.016 m$^2$ (10-12 cm), with differences among cases that can be considered minimal. Hence, to select the best hyper-parameter set, an arbitrary selection was performed, where for each hyper-parameter, the value that was more frequent within the five top-ranking combinations was selected. For instance, for the hyper-parameter number of neurons in Dense Layer 2 (Neurons Layer 2 , fourth column), the value *L*/2 was present four out of five times when the sampling rate was 10 s, hence is used as the final parameter. Repeating the procedure leads to the preferred network model, which are highlighted in bold for each sampling rate.

As expected, the shorter sampling rate yields smaller errors, although just marginally, suggesting that using subsampled series could have suffice. However, the longer sampling rate is compensated by smaller kernel sizes and stride, thus smaller neurons to perform feature identification. The reduced number of input data also results in fewer neurons, i.e., less overall parameters to be determined. Interestingly, the longer sampling rate favors the shallower offshore buoys, which can be indicative of these carrying more information than the deeper ones.

The hyper-parameter combinations of choice, shown in bold, are then used during training, where the actual network parameters are found. As before, the *MSE* is used as the primary metric to assess performance as shown in the last column of Table 3. The *MSE* training results improve upon those of validation.

It is of note that these results encompass all validation and training data, that were designed to have a class balance between inundating and non inundating scenarios. To investigate further how the networks are performing, the same metrics are computed separately distinguishing between scenarios that do and do not inundate in Table 4, which can be considered the case of maximum class imbalance. For the non inundating cases, typical *MSE* error values again range 1-2 cm, globally, and the *G* values are very small, indicating good correspondence between target and predicting time series. This shows that non inundating cases are well recovered, and that most of the overall error comes from the inundating cases, which can now reach up to $MSE=0.0595$ m$^2$ (24 cm), while errors in the case of no inundation are of the order $10^{-5}$ m$^2$. *G* also shows an increase but the maximum value is $G=0.2547$, indicating good predictions. For the case of inundating scenarios, the error for the maximum flow depth reaches up to $E_{d,i}=63$ cm, whereas the arrival time can be offset up to $E_{t,i}\sim$ 13 min.

Finally, the best performing networks (shown in bold in Table 4) are used to model the test data set, that is, cases not used before. The results of testing are summarized in Table 5. The performance of the networks is similar during validation, training and testing in terms of *MSE* and *G* values, and errors in amplitude and arrival time.

**Table 5:** Perfomance metrics test set.

Network	*MSE* Test		*G* Test		Test		False Alarm			Missed Alarm		
	Eq. (4)		Eq. (5)									
	Inund.	No Inund.	Inund.	No Inund.	$E_{d,i}$	$E_{t,i}$						
Model	$[m^{2}]$	$[m^{2}]$	$[-]$	$[-]$	[*m*]	[*min*]	A	B	C	A	B	C
CoB-200-10	0.017	2.58 E-05	0.1645	0.0053	0.35	12.39	3	0	0	0	0	0
LSB-50-60	0.007	8.01E-07	0.2525	0.0065	0.19	9.75	5	0	0	17	0	0
ViB-50-60	0.017	1.38E-04	0.0730	0.0258	0.22	1.57	16	5	0	0	1	0
VaB-50-60	0.044	2.95E-05	0.2126	0.0098	0.38	6.36	8	1	0	4	3	0

**Figure 5:**
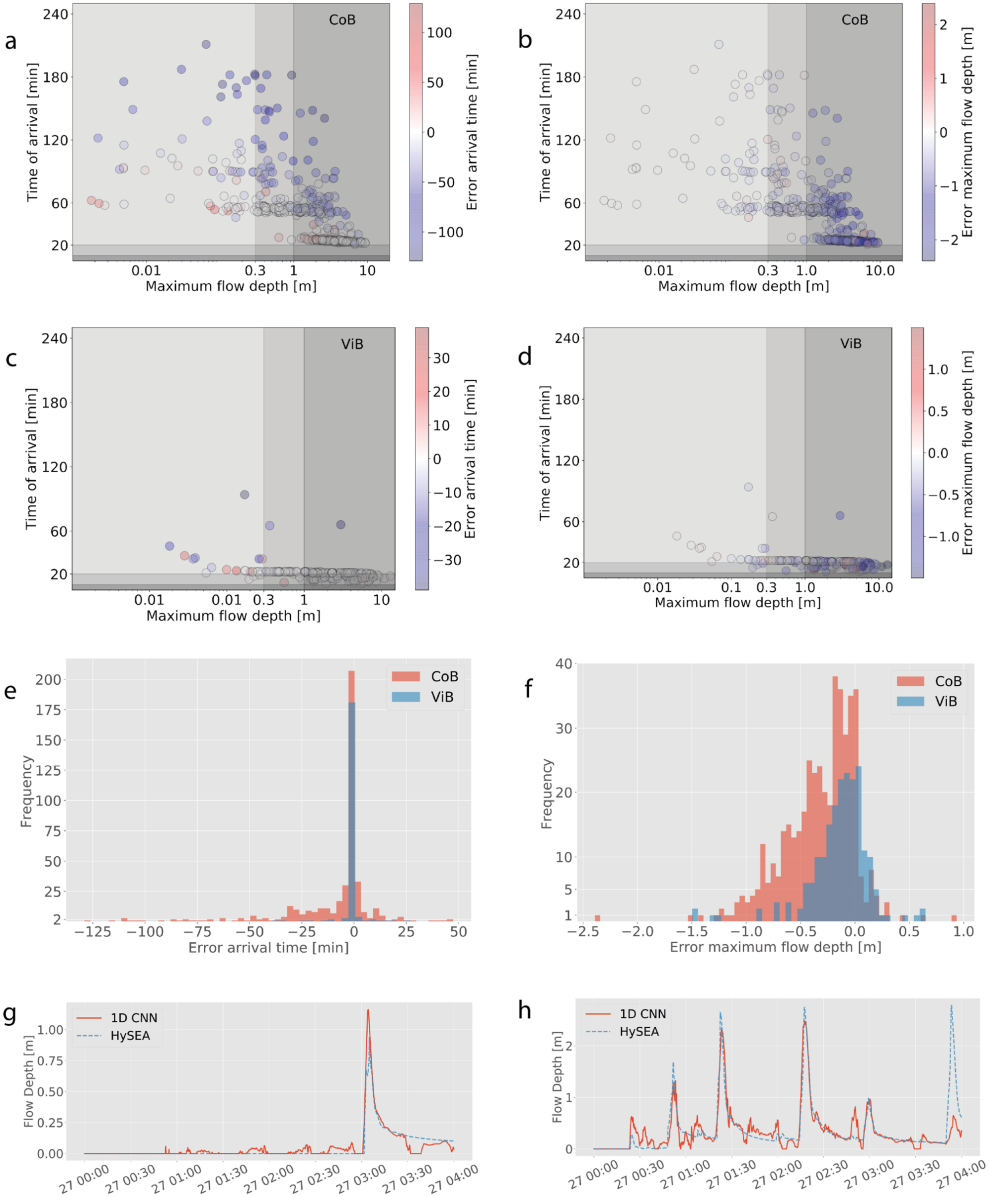
Error in arrival time $E_t$ (left column) and maximum flow depth $E_d$ for Coquimbo (CoB, top row) y Viña del Mar (ViB, second row). In (**a–d**) warmer colors indicate the NNM under estimates the hazard. (**e, f**) The histograms of each error among all cases. (**g, h**) The time series that lead to the maximum error, where the full forward model using Tsunami-HySEA is shown as dashed blue lines, and the NNM prediction in light red. (**g**) ViB, h) CoB.

Even though the absolute value of the errors seems large, the effect on the hazard categorization is minimal, even during testing when the NNM are used with data not seen before (Table 5, last two columns). Indeed, for inland gauge CoB, only three scenarios of the 1017 used in testing triggered false alarms, and these were only for the lower hazard level A . On the other hand, no scenario caused missed alarms and only six cases were missed alarm during validation (Table 4). As before, the errors were found only for the lower hazard level. The worst performing inland gauge on this regard was ViB for false alarms, where up to 21 scenarios showed false alarms during testing (2 %) and 93 during validation (9%), and LSB, for missed alarms. For the latter, up to 29 missed alarm ocurred during validation (3%) and 17 during testing (2%) The most critical hazard levels, B and C, that in case of the Chilean system are associated to evacuation, caused no more than 2 errors for missed alarms (0.02%). The reason for this behavior is illustrated in Fig. 5. In the top panels, the joint distribution of the errors in arrival time $E_t,i$ and flow depth $E_d,i$ are shown as circles, whereas the color scale indicates the value of the error. The colorscale has been intentionally made symmetric, hence actual data do not span the overall extent of it. The third row shows the corresponding histogram of the errors. While the error in arrival time $E_t$ can be large for relatively late arrivals (later than 60 min) in Coquimbo, most of the scenarios concentrate arrivals within a few minutes. The error $E_t$ is biased negative, meaning earlier arrival in the NNM. While less than ideal, this can be an unintended conservative feature within the context of early warning. The reason for these early arrivals is shown in the sample time series in the bottom row, where data with the worst $E_d$ are shown. Small scale fluctuations are predicted by the NNM, which trigger early detection and drive errors in $E_t$. Errors in flow depth $E_d$ are also biased negative and can reach up to 2.5 m. Despite the large value, these occur typically when actual flow depths exceed 3–4 m (see how data clusters towards high flow depth in Fig. 5b,d, noting that the horizontal scale is logarithmic). Hence, these errors do not change the hazard categorization. Second, the timing of the maximum value is retained in Eq. (6) which could affect a few cases, as shown in Fig. 5h, which shows the wave with largest error ($E_{d,i}\approx -2.5$ m, see Fig. 5f). This is an extreme situation where the maximum flow depth ($max\{d^{HR}\})$ occurs nearly 3 h after first arrival and is concurrent with a poor NNM prediction. However, the hazard level was characterized by three large waves earlier in the time series which were well predicted. Even in cases like these, the overall temporal structure is well recovered by the NNM up until that point, even when several inundation phases occur. This is sustained by the low values of *G*. In Fig. S7 of the Supplemental Material, histograms of *MSE* and *G* are shown. The low values suggest that the NNMs perform well in predicting the time series.

**Figure 6:**
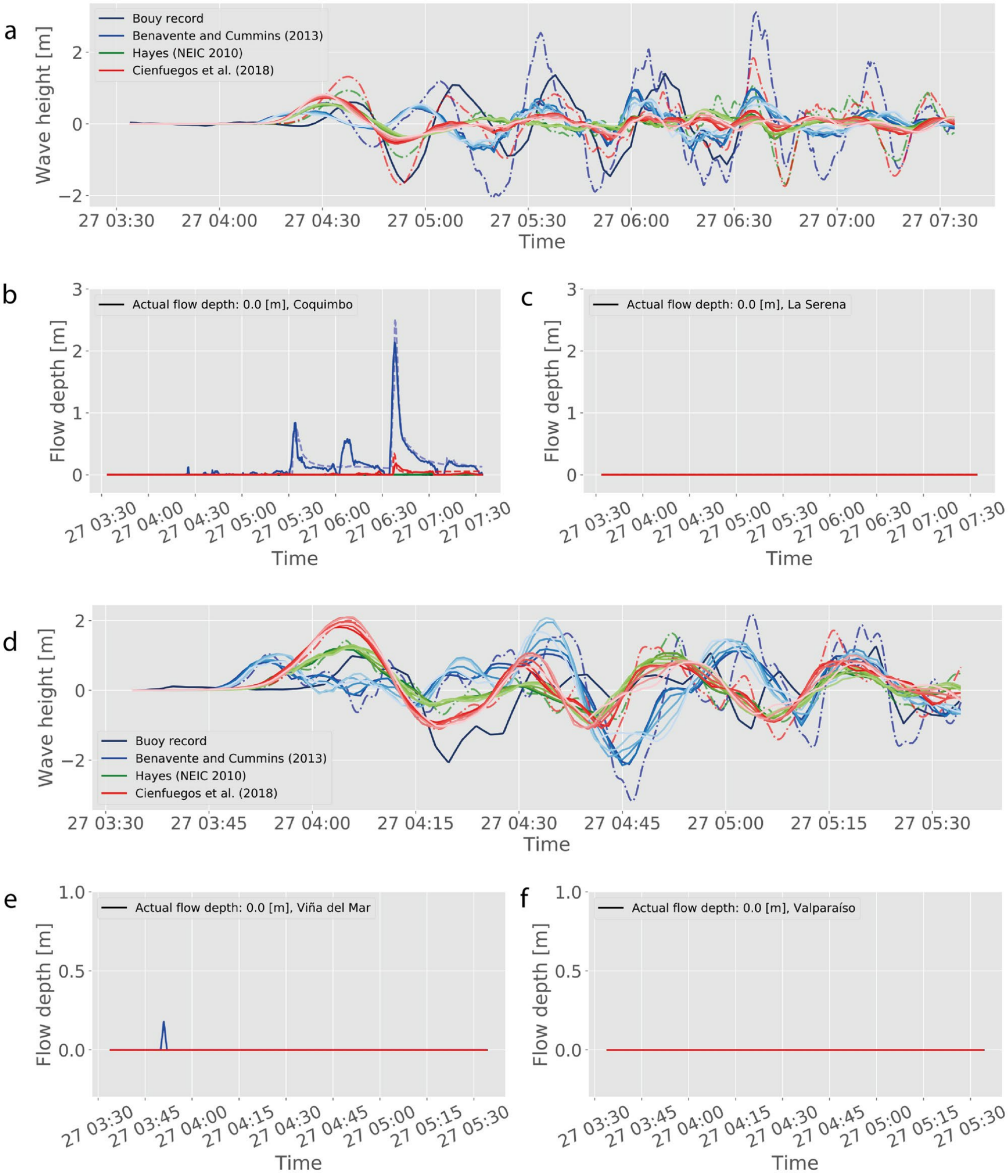
Hazard assessment for the Maule 2010 event. (**a, d**) The offshore tsunami time series at each of the six input buoys when modeled using as source condition that of Benavente and Cummins^72^ (blue lines), Hayes (NEIC, 2010) taken from the SRCMOD database^71^ (green lines) and the median model of Cienfuegos et al.^13^. The black line shows the nearest tide gauge record. Dashed lines correspond to the high-resolution tsunami HySEA model at the location of the tide gauge. (**b, c**) and (**e, f**) The time series of inundation as predicted by the corresponding network models and tsunami HySEA (dashed). Top panels are for Coquimbo-La Serena, and bottom panels correspond to Valparaíso-Viña del Mar.

**Figure 7:**
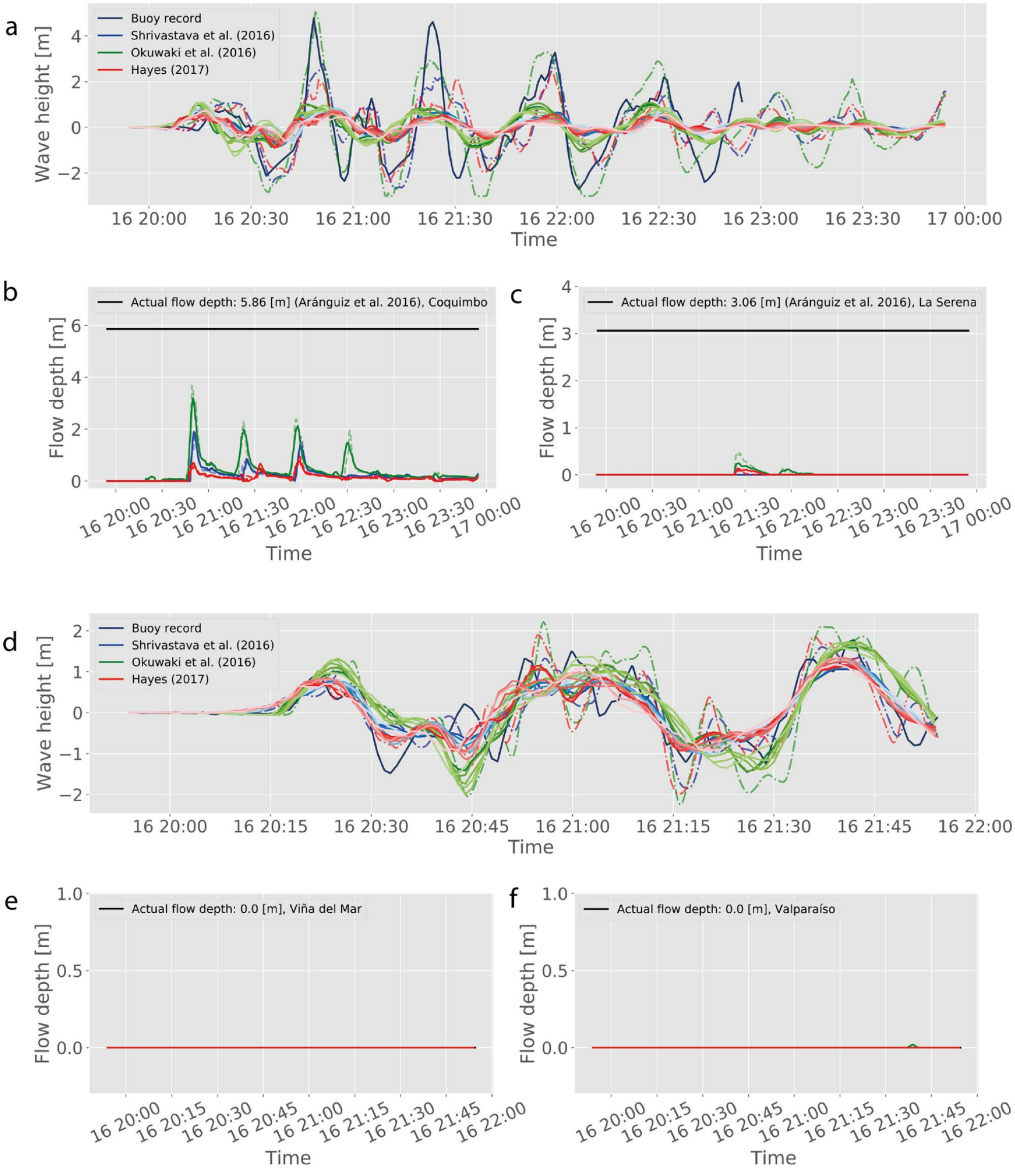
Results for the Illapel 2015 event. Same key as Fig. 6, with the exception of blue lines corresponding to Shrivastava et al.^74^, green lines to Okuwaki et al.^73^, and red lines correspond to Hayes^75^ finite fault model (SRCMOD^71^ and reference therein).

Figures 6 and 7 show the results of using the NMMs to predict the outcome of the historical tsunamis. For the Maule 2010 event, most of TIMs and hazard assessments match the in situ observations, when no inundation was observed in either LSB, ViB nor VaB. Hence, the NNMs of these locations are able to reproduce successfully the case of no alarm. However, the situation is different for CoB, where the NNM predicts inundation of up to 2 m, which would have prompted evacuation, whereas no actual inundation did occur, thus a false alarm. It is of note, that this happened only for the Benavente and Cummins^72^ source solution, whereas the median model of Cienfuegos et al.^13^ predicts a small inundation that did not exceed the lowest threshold (hence no alarm), and the Hayes (NEIC 2010)^71^ source yields zero inundation. In the case of the Illapel 2015 event, again ViB and VaB perform well, successfully predicting no inundation. However, the situation is more complex for the Coquimbo-La Serena region. While the CoB NNM predicts inundation at a level large enough to have prompted evacuation (hazard properly categorized), none of the network models is capable to predict the measured flow depth of nearly 6 m^60,62,90^. Notably, the Hayes model^75^ for the Illapel event forecasts very small flow depths (a missed alarm). LSB, on the other hand, is a case of missed alarm as none of the models predicts flow depths that match the observed 3 m.

## Discussion

From the results from testing, it can be seen that the NNMs do a good job in predicting the outcomes of possible inundation among the synthetic data set. The overall design of a network requires short computational times. For example, considering the same hyperparameters, training of the 2,234,213 parameters requiered for CoB at 10 s required approximately 10 min, whereas for the 242,613 parameters of CoB at 60 s, took 3.5 min, roughly a 3X speed up. This could enable scaling up the process to multiple gauges at minimal cost, yet allowing for differences in the NMMs. Moreover, the time required to make a prediction is of about 1 s in an off-the-shelf Quadcore laptop with Intel Core i7-6600U CPU at 2.60 GHz, running Ubuntu 18.04.5, making it suitable for TEWS temporal requirements. For comparison, full forward modeling of inundation using Tsunami-HySEA with two Nvidia Tesla V100/16GB-HBM2 GPU cards took up to 10 min.

The final NNM varied among the target locations. Notably, some of the locations yield better performance using the subsampled time series and with offshore buoys located at 50 m water depths. This was the case of ViB,VaB, and LSB. In contrast, CoB worked best with inputs at 200 m but the higher sampling rate, which is an apparent trade off between the sampling rate and the possible non linearity of the tsunami wave in shallower water. CoB has some characteristics that set it apart from the others. First, it is located on a zone where actual tsunamis have inundated in the past, and a larger fraction of the modeled tsunamis produced inundation. This could mean that the inundation characteristics varied among scenarios enough to require denser input data to distinguish among them. Alternatively, non linear processes such as resonance have been identified in the area, and it can be speculated that the deeper water buoys were more stable in terms of input. Regardless of the actual explanation, what is relevant for the purposes of this implementation, is that the use of distinct target inland gauges even for neighboring locations can be recommended, because they could be trained with smaller data sets and leaner NNM than a more complex, one-for-all inundation mapping network.

Regarding the predictive capability of the NMMs, the results appear to indicate that at times, large mismatch with in situ data could occur. While this could have been seen as a failure of the proposed approach, this is not the case. Careful inspection of results shows that even the high-resolution modeling using Tsunami-HySEA is not capable of matching the observations, as shown by the dashed lines in Figs. 6 and 7. Moreover, the hazard assessment is essentially the same that would have been obtained if the full forward modeling runs were used instead. This suggests that the problem lies in either the initial conditions and/or the boundary conditions, and not in the NNM capability.

This highlights the challenges of accurately predicting inundation rather than shortcomings in the predictive capability of the proposed NNM. For instance, the CoB buoy is located near the area of maximum inundation and amplification of the tsunami due to resonance in Coquimbo bay which may not be well reproduced even with the full forward modeling. In addition, the variability of results among the sources tested also suggest a source dependency. These effects imply a significant challenge for a TEWS, because it would force it to consider source variability and a large number of source scenarios and their corresponding assessments to develop a measure of the uncertainty^13,15^. The use of 1D CNN networks can aid on this regard, as these surrogate models can offer similar predictive capabilities at a minimal computational cost. Of course, this still requires very fast propagation modeling to feed the network. However, this already can be achieved in times adequate for early warning^15^.

Another explanation is that the NNM were overfitting their response to the high resolution models. However this is considered not to be the case, as neither the Illapel 2015 nor Maule 2010 source models were used in training or testing. Moreover, the Maule 2010 main rupture zone is located south from the scenario generation zone used in training, meaning that they could not have been seen at all by the network beforehand. Hence, rather than limiting the applicability of the NNM to events generated only in this region, these results suggest it could be applied to other regional sources even if they were not included in the network design phase. However, more testing should be done on this regard.

These results also indicate that the methodology can capture different hydrodynamic behavior. It was found that most inundating tsunamis in the region of Valparaíso-Viña del Mar had a temporal structure characterized by a large first inundating wave (cf. time series in Fig. 2), followed by trailing waves that often did not exceed the first. Hence, this region appears to be more susceptible to inundation to the first packet of tsunami energy. Coquimbo-La Serena are susceptible to resonance, where larger waves can develop later and even after smaller early inundation phases. Overall, the NNMs were able to reproduce these behaviors with small errors in both mean statistics, and hazard assessments.

## Conclusions

The present work aims to assess the possibility and capabilities of learn 1D CNN networks to be used in Tsunami Early Warning. Rather than attempting to estimate a unique network that could map inundation at a large number of points, the focus was set on neural network models (NNM) designed to reproduce time series of tsunami inundation at specific locations. The procedure can be applied to several independent locations to increase coverage, if needed, at small computational cost.

In addition, the design of the NNM considered the analysis of the tsunami inundation characteristics using high resolution data, which allowed to perform a pre-processing of the time series that contributed to reduce the training burden. For generality, the method was tested at four specific locations on two bays that differ in their hydrodynamic response. The results showed a high level of success in predicting the inundation characteristics, with the ability to distinguish among scenarios that inundate and those that do not, an essential feature for TEWS. This was true when compared with synthetic data not seen by the network before. However, when tested against actual tsunamis, one case of a false alarm and one case of a missed alarm were found. Careful inspection showed that the network models were capable of matching high resolution modeling results, suggesting the origin of error was elsewhere. These errors would affect any modeling exercise, and were not associated to the methodology presented here.

With these considerations, the proposed approach offers a cost-efficient alternative to provide a surrogate for inundation time series within the context of Tsunami Early Warning time windows. While accurate Near Real Time modeling still appears to be the most reliable choice, the presence of significant uncertainties in source characterization, bathymetry and other sources of uncertainty may require a large number of simulations that can exceed the allocated times. The use of surrogate models may allow to provide multiple assessments within reasonable times, with a small trade-off in accuracy. It is proposed that these simple NNMs can be up to this task. More work needs to be done, however, to ensure that these type of surrogate models do not introduce excessive uncertainty into an already uncertain predictive scheme. This will be a subject of subsequent work.

## Supplementary Information

Supplementary Information.
