# Supplementary material for: Discriminating the occurrence of inundation in tsunami early warning with one-dimensional convolutional neural networks
Source: Sci Rep. 2022 Jun 20;12:10321. doi: 10.1038/s41598-022-13788-9 (PMC9209464; doi:10.1038/s41598-022-13788-9)
Supplement: Supplementary file 1 — Supplementary Information. [file 41598_2022_13788_MOESM1_ESM.pdf]

Supplementary Material: Discriminating the Occurrence of Inundation in Tsunami Early Warning with One-Dimensional Convolutional Neural Networks.

Jorge Núñez[1], Patricio A. Catalán[1,*], Carlos Valle[2], Natalia Zamora[3], and Alvaro Valderrama[4]

[1]Departamento de Obras Civiles, Universidad Técnica Federico Santa María, Valparaíso, 2390123, Chile
[2]Departamento de Ciencia de Datos e Informática, Universidad de Playa Ancha Valparaíso, Chile
[3]Computer Applications in Science and Engineering Department, Barcelona Supercomputing Center (BSC), 08034 Barcelona, Spain
[4]Universidad Técnica Federico Santa María, Valparaíso, Chile
[*]patricio.catalan@usm.cl

ABSTRACT

In this supplementary material, a brief explanation of the procedure to construct the seismic scenario database is explained. These scenarios are used as input data for the tsunami numerical model Tsunami-HySEA at both low and high resolution, which are then used in the design of the one-dimensional convolutional neural network model (1D CNN). Additionally, two complementary plots for the distribution of the training, testing and validation cases, and performance metrics are included.

1 Input data for the 1-CNN. Stochastic seismic sources

In this study, a database of 6776 scenarios with magnitudes in the range M_w 8.0-9.2 has been generated. These scenarios are the initial condition used to model tsunami inundation using Tsunami-HySEA[1] with four-level nested grids with higher resolutions in the coastal cities of Valparaíso, Viña del Mar, La Serena and Coquimbo.

For the present implementation, the first step is to define a geometry along where synthetic (stochastic) seismic sources will be generated. Here, the geometry of megathrust seismogenic zone is within the mid-southern portion of the Zone 2 (Z2) in Poulos et al[2], as shown with ZV in Figure 1 (main text). This segment has been discretized in 1418 subfaults of 10 km x 10 km, having enough resolution to resolve the slip distribution. For this task, the MudPy open-source code[3,4] has been used.

For the set of magnitudes M_w 8.0-9.2, ruptures size require scaling relations that could transform magnitudes to areas[5], more specifically length and width. The MudPy code generates a variable slip pattern distribution assuming a normal distribution of the slip on each subfault, with a vector containing the slip for each realization limited to positive values using an exponential of the normal distribution, this is, a log-normal distribution. The resulting slip distribution has a mean vector (μ) and a covariance matrix that are a fraction of the mean slip and a rupture correlation function. As shown by Leveque *et al.*[6], these parameters control the spatial statistics of slip variability. This generation of the stochastic seismic scenarios is similar to the approach described in Zamora *et al.*[7], assuming a maximum slip truncated at $\sim$ 50 m, which corresponds to a larger value of the expected geodetic slip deficit of the central Chile Region[8] (ZV geometry). This region is of interest because it corresponds to an area where large asperities have been identified[9], and corresponds with one of the suggested rupture areas of the 1730 M_w 9.1-9.3[10]. This area has been also used in a probabilistic tsunami hazard assessment in central Chile[11].

Tsunami numerical simulations are done using the Tsunami-HySEA code[1]. Tsunami-HySEA solves the 2D nonlinear one-layer shallow water system in both spherical and Cartesian coordinates, based on a finite volume method. Here, the spherical-coordinates version is used. The Okada analytical equations[12] are implemented to obtain

the ground deformations, and therefore, sea level initial condition for the numerical simulations. More information can be found at https://edanya.uma.es/hysea.

The following figures show the summary of statistics related to the generation of the input data which are finite fault seismic sources. These data are used for the testing and training of the 1-CNN. Figure S1 shows the location of the centroids of 6776 ruptures in the range of magnitudes M_w 8.0-9.2 (small cyan triangles). The location of the centroids are generated during the first step of the Mudpy[3] procedure. The location of the centroids of the two historical events are shown as large cyan triangles, taken from the Global Centroid Moment Tensor (GCMT)[13].

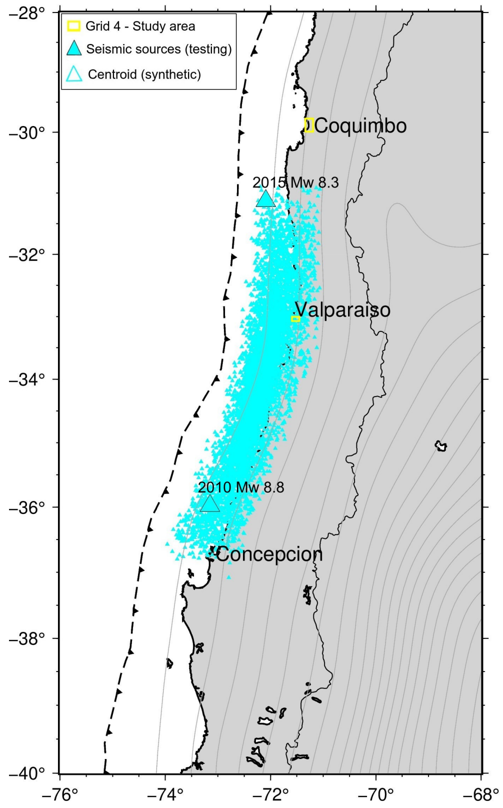

Figure S1. Centroid location of synthetic data and real cases used for testing the algorithms. The slab contours (in grey) are taken from Hayes *et al.*[14].

As an estimate of the variability among scenarios, the slip distribution for the 6776 seismic scenarios are analyzed in unison with different statistical metrics, as shown in Figure S2. Figure S2a) shows some areas of large slip up to the maximum value, but without any significant structure suggesting low correlation. The 95% percentile (Fig. S2b) shows a significant decrease in its value, that is nearly a half of the peak value. It shows a tendency to concentrate larger slip towards the shallower section of the interface, which is considered a conservative situation as shallower events should lead to larger tsunamis[15]. The median slip is well distributed, with a small variation in magnitude (Fig. S2c,d). These results suggest a good areal coverage and distribution of the slip among the 6776 sources, thereby providing a reasonable range of tsunami conditions.

To further explore the database, Figures S3 and S4 show the distribution of all events in terms of their length and width, as a function of moment magnitude. Length and width were estimated from each scenario as the distance between the extreme non-zero slip locations in the along strike and along dip directions, respectively. These distributions compare well with those presented by De Risi and Goda[16], for instance. It can be noted that the width saturates at large magnitude, which is a result of the finite along dip extent of the rupture zone. This saturation induces either larger slip and/or longer ruptures[17].

Figure S5 shows the finite fault models of the six historical source solutions used for testing. These are three

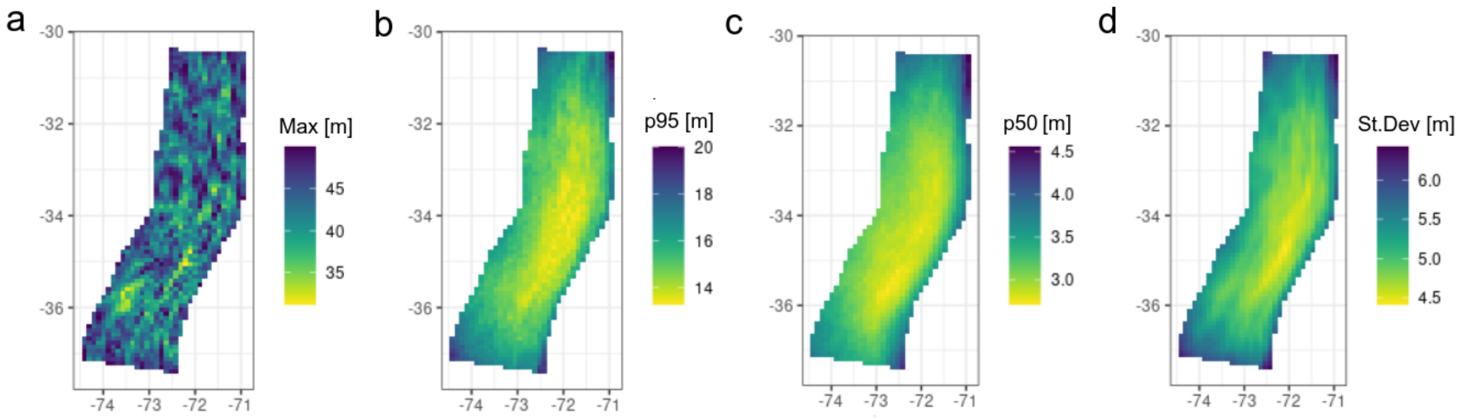

Figure S2. Slip distribution statistics. a. Maximum values; b. Percentile 95%; c. Percentile 50 %; d. Standard Deviation. Zero slip values in each cell treated as NaN. Stochastic slip distribution generation using the Mudpy code[4].

finite fault models from inversions for the 2010 Mw 8.8 Maule earthquake: Benavente and Cummins[18], the model from Hayes (NEIC 2010) taken from the SRCMOD database[19] and the median value obtained by Cienfuegos et al.[20]. For the 2015 Mw 8.3 Illapel earthquake, those from Okuwaki et al.[21], Hayes[22] and Shrivastava et al.[23].

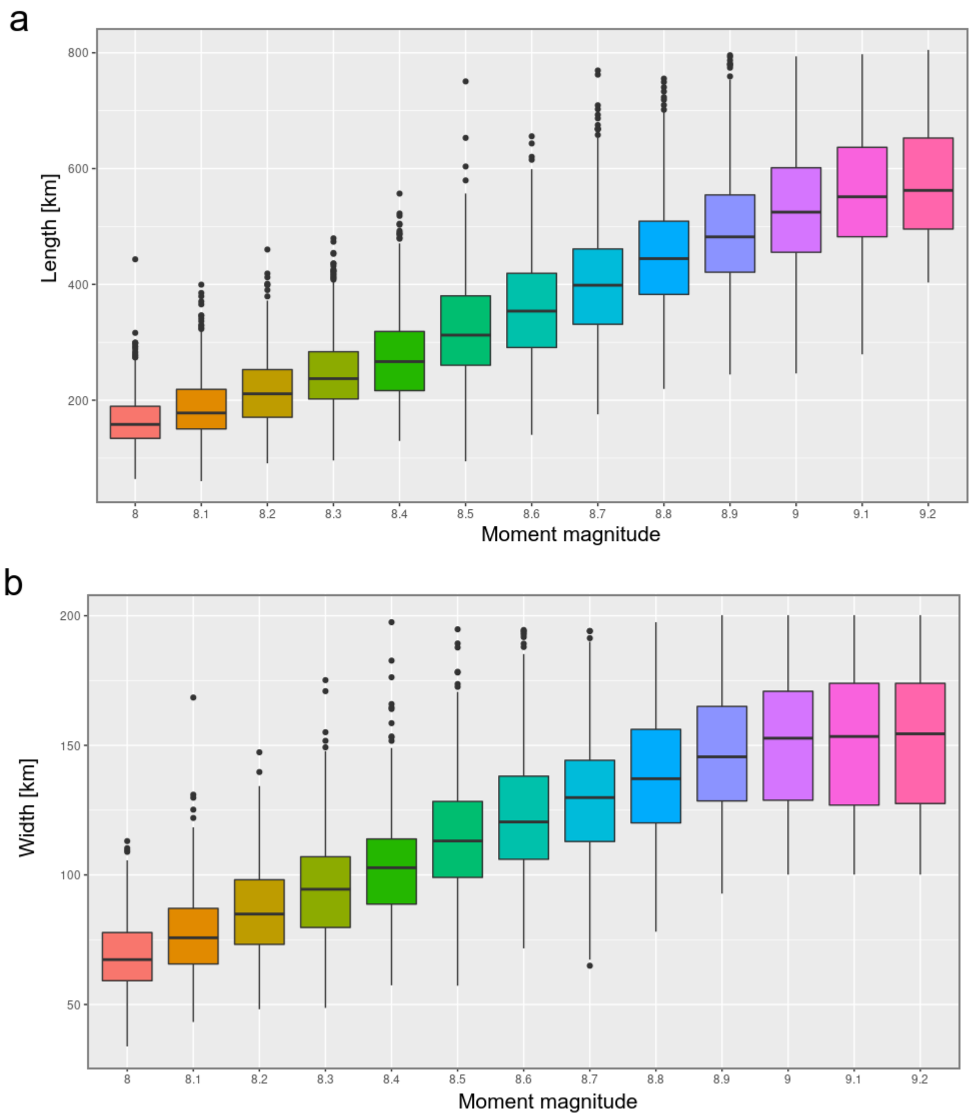

Figure S3. Relation of magnitude and a. Along strike length and b. Along dip width of the synthetic ruptures shown as boxplot functions of moment magnitude.

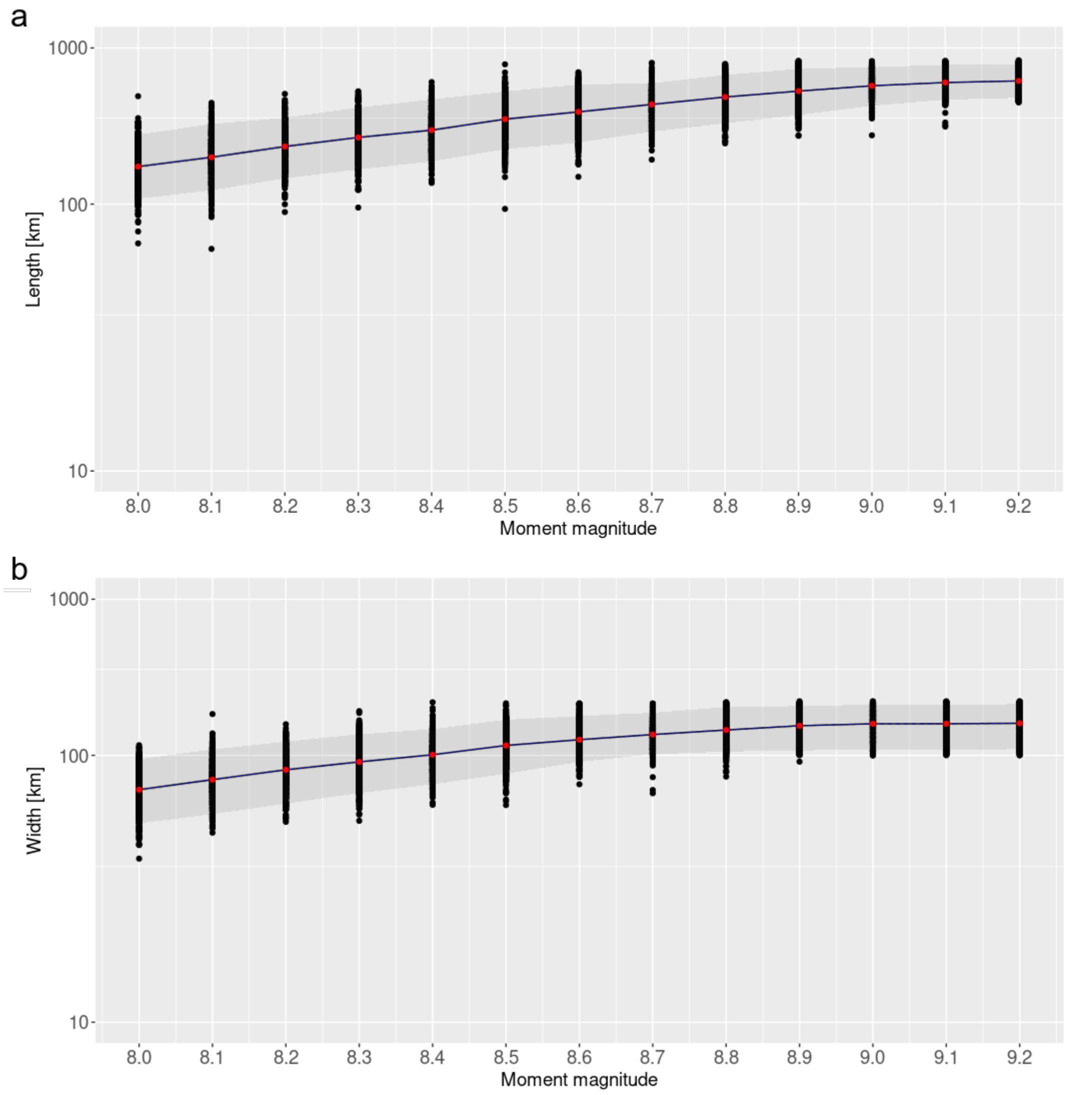

Figure S4. Confidence values of the magnitude and a. length and b. width of the synthetic ruptures. Blue line with red points show the central mean values for those two parameters in each moment magnitude bin.

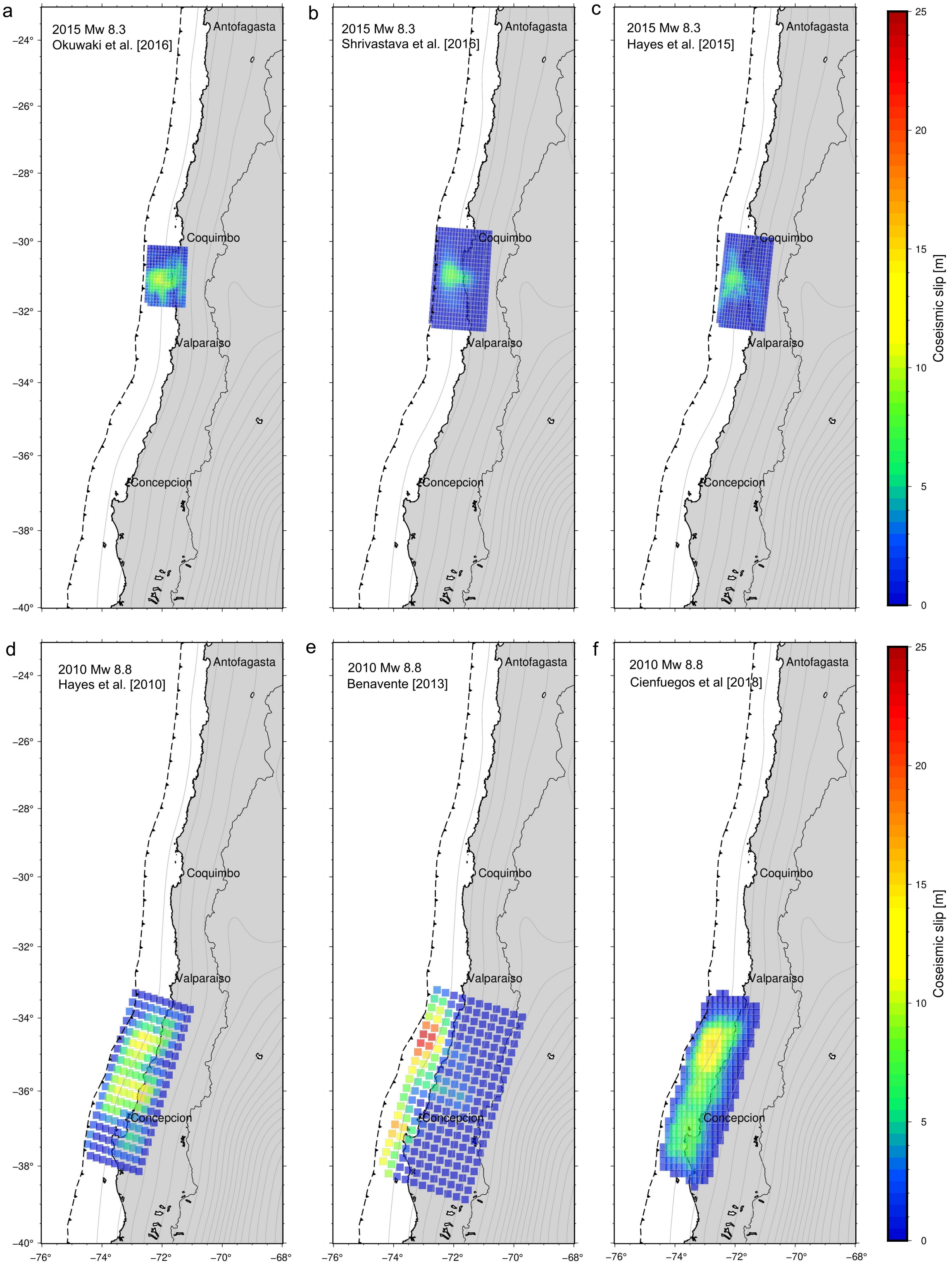

Figure S5. Finite fault models used for testing the algorithmn. a. Illapel (Coquimbo) 2015 Mw 8.3 earthquake, model from Okuwaki et al.[21]; b. Illapel (Coquimbo) 2015 Mw 8.3 earthquake, model from Shrivastava et al.[23]; c. Illapel (Coquimbo) 2015 Mw 8.3 earthquake, model from Hayes[22] retrieved from SRCMOD[19]; d. Maule 2010 Mw 8.8 earthquake, model from Benavente and Cummins[18]; e. Maule 2010 Mw 8.8 earthquake, model from Hayes (NEIC 2010) taken from[19]; f. Median of the slip of the Maule Mw 8.8 earthquake that resulted from the integration of 19 finite fault models by Cienfuegos et al.[20]. Most of the models can be found at the SRCMOD database[19].

2 Complementary Figures

In the main text, a sample figure is presented to show the distribution of scenarios among the training, validation and testing data sets. Here, Figure S6 shows the same plots for the remainder cases are presented for completeness. It can be noted that in all cases, the distributions are similar along the cases. The most noticeable differences occur when flow depths are very small, which does not have an impact on the hazard categorization. On the other hand, the training data sets (red lines) typically reach larger extreme values for flow depth, which reduces the possibility of extrapolation.

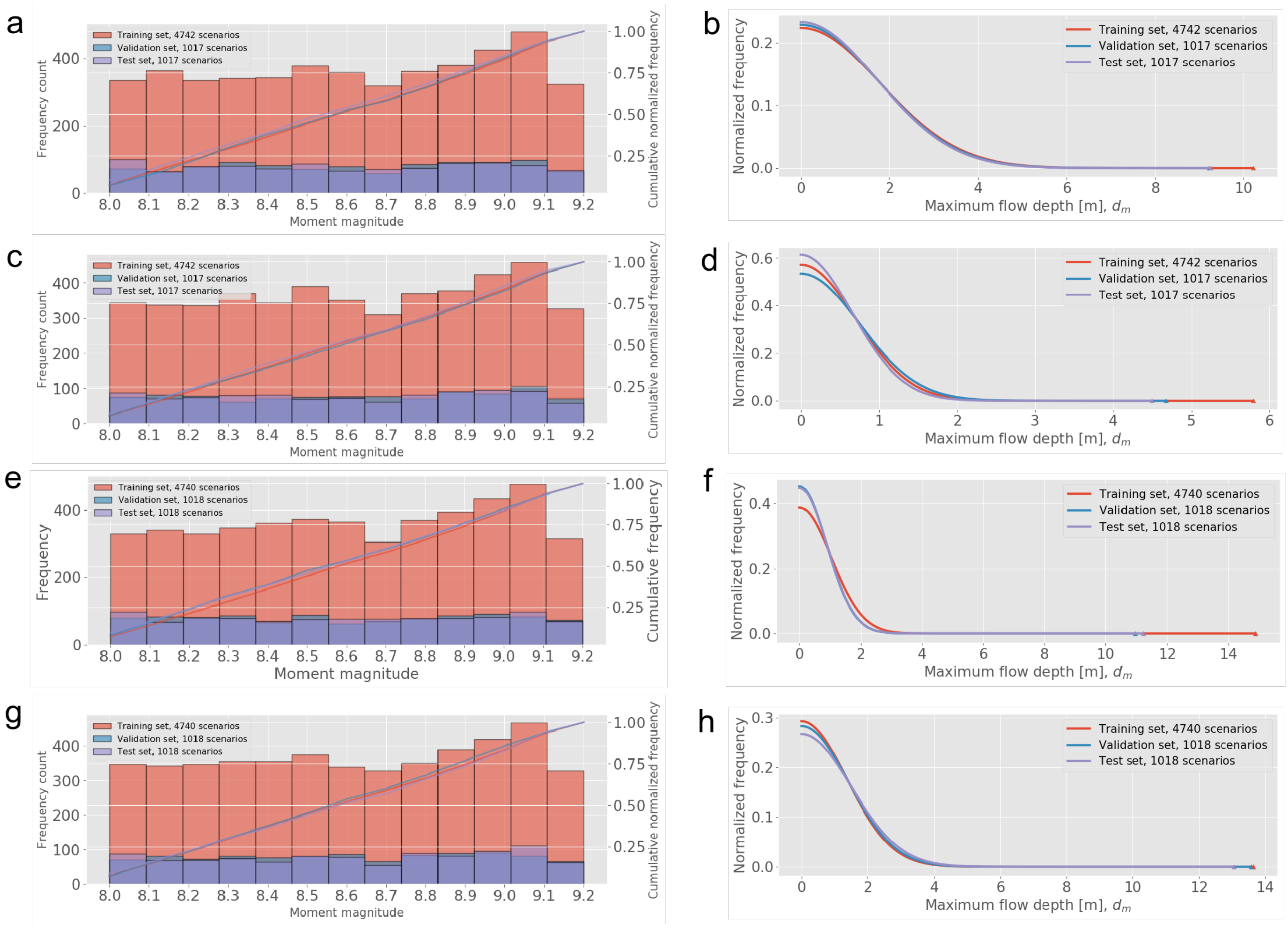

Figure S6. Statistics of the distribution of scenarios among validation, training and test data sets for a-b) Coquimbo (CoB), c-d) La Serena (LSB), e-f) Valparaíso (VaB) and g-h) Viña del Mar (ViB). Left column plots show the distribution of scenarios as function of moment magnitude (bars) and normalized cumulative frequency. Right column plots show the frequency distribution in terms of maximum flow depth $max\{d^{HR}\}$. Symbols denote the extrema of each set.

Finally, in Fig. S7, the histograms of the performance metrics on the testing data set are shown for CoB and ViB. It can be seen that most of the MSE and G are very small, indicating a good accuracy in reproducing the time series.

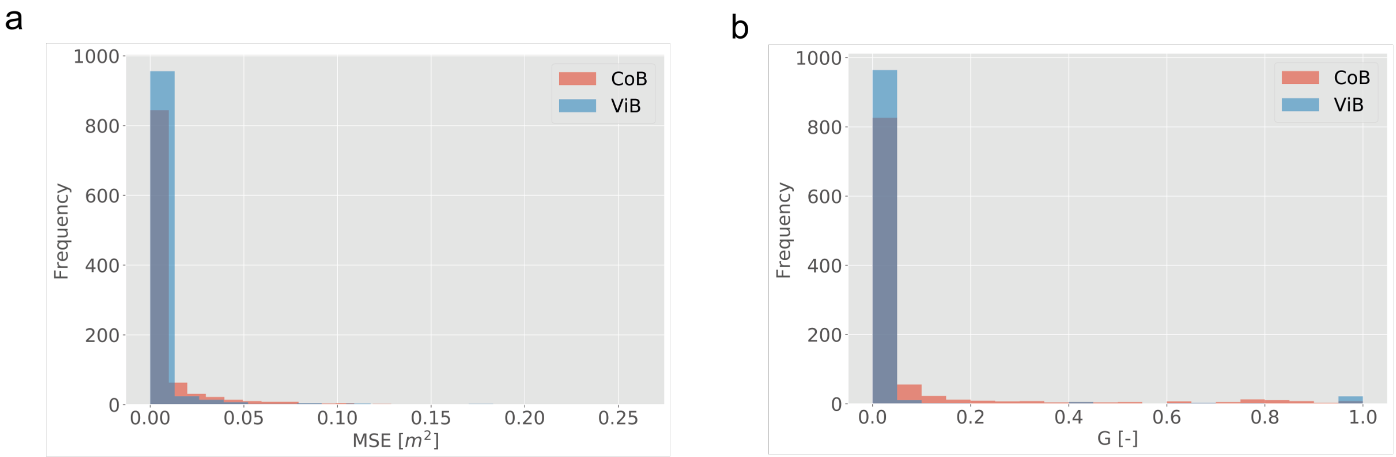

Figure S7. Histograms of the performance metrics on the testing data set are shown for CoB and ViB. a) MSE, b) G.

References

1. Macías, J., Castro, M. J., Ortega, S., Escalante, C. & González-Vida, J. M. Performance Benchmarking of Tsunami-HySEA Model for NTHMP's Inundation Mapping Activities. *Pure Appl. Geophys.* DOI: 10.1007/s00024-017-1583-1 (2017).

2. Poulos, A., Monsalve, M., Zamora, N. & de la Llera, J. C. An Updated Recurrence Model for Chilean Subduction Seismicity and Statistical Validation of Its Poisson Nature. *Bull. Seismol. Soc. Am.* **109**, 66–74, DOI: 10.1785/0120170160 (2019).

3. Melgar, D. & Bock, Y. Kinematic earthquake source inversion and tsunami runup prediction with regional geophysical data. *J. Geophys. Res. Solid Earth* **120**, 3324–3349, DOI: 10.1002/2014JB011832 (2015). 2014JB011832.

4. Melgar, D., LeVeque, R. J., Dreger, D. S. & Allen, R. M. Kinematic rupture scenarios and synthetic displacement data: An example application to the cascadia subduction zone. *J. Geophys. Res. Solid Earth* **121**, 6658–6674, DOI: 10.1002/2016JB013314 (2016). 2016JB013314.

5. Melgar, D. & Hayes, G. P. The correlation lengths and hypocentral positions of great earthquakes. *Bull. Seismol. Soc. Am.* **109**, 2582–2593, DOI: 10.1785/0120190164 (2019).

6. LeVeque, R. J., Waagan, K., González, F. I., Rim, D. & Lin, G. Generating Random Earthquake Events for Probabilistic Tsunami Hazard Assessment. *Pure Appl. Geophys.* **173**, 3671–3692, DOI: 10.1007/s00024-016-1357-1 (2016).

7. Zamora, N., Catalán, P. A., Gubler, A. & Carvajal, M. Microzoning tsunami hazard by combining flow depths and arrival times. *Front. Earth Sci.* **8**, DOI: 10.3389/feart.2020.591514 (2021).

8. Métois, M., Vigny, C. & Socquet, A. *The Chile-2015 (illapel) Earthquake and Tsunami. Pageoph Topical Volumes.*, vol. 173, chap. Interseismic Coupling, Megathrust Earthquakes and Seismic Swarms Along the Chilean Subduction Zone (38–18S), 45–63 (Springer International Publishing, Cham, 2017).

9. Sippl, C., Moreno, M. & Benavente, R. Microseismicity Appears to Outline Highly Coupled Regions on the Central Chile Megathrust. *J. Geophys. Res. Solid Earth* **126**, DOI: 10.1029/2021JB022252 (2021).

10. Carvajal, M., Cisternas, M. & Catalán, P. A. Source of the 1730 Chilean Earthquake from Historical Records: Implications for the Future Tsunami Hazard on the Coast of Metropolitan Chile. *J. Geophys. Res. Solid Earth* **122**, 3648–3660, DOI: 10.1002/2017JB014063 (2017). 2017JB014063.

11. Becerra, I., Aránguiz, R., González, J. & Benavente, R. An improvement of tsunami hazard analysis in Central Chile based on stochastic rupture scenarios. *Coast. Eng. J.* **62**, 473–488, DOI: 10.1080/21664250.2020.1812943 (2020).

12. Okada, Y. Surface deformation due to shear and tensile faults in a half-space. *Bull. seismological society Am.* **75**, 1135–1154 (1985).

13. Ekström, G., Nettles, M. & Dziewoński, A. The global CMT project 2004–2010: Centroid-moment tensors for 13,017 earthquakes. *Phys. Earth Planet. Interiors* **200–201**, 1–9, DOI: 10.1016/j.pepi.2012.04.002 (2012).

14. Hayes, G. P. *et al.* Slab2, a comprehensive subduction zone geometry model. *Science* **362**, 58–61, DOI: 10.1126/science.aat4723 (2018).

15. Geist, E. L. Complex earthquake rupture and local tsunamis. *J. Geophys. Res.* **107**, 2086, DOI: 10.1029/2000JB000139 (2002).

16. De Risi, R. & Goda, K. Probabilistic Earthquake–Tsunami Multi-Hazard Analysis: Application to the Tohoku Region, Japan. *Front. Built Environ.* **2**, 25, DOI: 10.3389/fbuil.2016.00025 (2016).

17. Davies, G. Tsunami variability from uncalibrated stochastic earthquake models: Tests against deep ocean observations 2006–2016. *Geophys. J. Int.* **218**, 1939–1960, DOI: 10.1093/gji/ggz260 (2019).

18. Benavente, R. & Cummins, P. R. Simple and reliable finite fault solutions for large earthquakes using the W-phase: The Maule (Mw = 8.8) and Tohoku (Mw = 9.0) earthquakes. *Geophys. Res. Lett.* **40**, 3591–3595, DOI: 10.1002/grl.50648 (2013).

19. Mai, P. M. & Thingbaijam, K. K. S. SRCMOD: An online database of finite-fault rupture models. *Seismol. Res. Lett.* **85**, 1348–1357, DOI: 10.1785/0220140077 (2014).

20. Cienfuegos, R. *et al.* What can we do to forecast tsunami hazards in the near field given large epistemic uncertainty in rapid seismic source inversions? *Geophys. Res. Lett.* **45**, 4944–4955, DOI: 10.1029/2018GL076998 (2018).

21. Okuwaki, R., Yagi, Y., Aránguiz, R., González, J. & González, G. Rupture Process During the 2015 Illapel, Chile Earthquake: Zigzag-Along-Dip Rupture Episodes. *Pure Appl. Geophys.* 1–10, DOI: 10.1007/s00024-016-1271-6 (2016).

22. Hayes, G. P. The finite, kinematic rupture properties of great-sized earthquakes since 1990. *Earth Planet. Sci. Lett.* **468**, 94–100, DOI: 10.1016/j.epsl.2017.04.003 (2017).

23. Shrivastava, M. N. *et al.* Coseismic slip and afterslip of the 2015 mw 8.3 illapel (chile) earthquake determined from continuous GPS data. *Geophys. Res. Lett.* **43**, 10,710–10,719, DOI: 10.1002/2016GL070684 (2016).
